# Musashi-2 in cancer-associated fibroblasts promotes non-small cell lung cancer metastasis through paracrine IL-6-driven epithelial-mesenchymal transition

**DOI:** 10.1186/s13578-023-01158-5

**Published:** 2023-11-08

**Authors:** Parinya Samart, Gayathri Heenatigala Palliyage, Surapol Issaragrisil, Sudjit Luanpitpong, Yon Rojanasakul

**Affiliations:** 1 Department of Pharmaceutical Sciences, West Virginia University, Morgantown, WV 26506 USA; 2 Siriraj Center of Excellence for Stem Cell Research, Faculty of Medicine Siriraj Hospital, Mahidol University, 2 Siriraj Hospital, Bangkoknoi, Bangkok, 10700 Thailand; 3 Division of Hematology, Department of Medicine, Faculty of Medicine Siriraj Hospital, Mahidol University, Bangkok, Thailand; 4 WVU Cancer Institute, West Virginia University, Morgantown, WV USA

**Keywords:** Musashi-2, Tumor microenvironment, Cancer-associated fibroblast, Non-small cell lung cancer, Metastasis, IL-6, Epithelial-mesenchymal transition

## Abstract

**Background:**

Lung cancer, the most common cause of cancer-related mortality worldwide, is predominantly associated with advanced/metastatic disease. The interaction between tumor cells and cancer-associated fibroblasts (CAFs) in tumor microenvironment is known to be essential for regulating tumor progression and metastasis, but the underlying mechanisms, particularly the role of RNA-binding protein Musashi-2 (MSI2) in CAFs in promoting non-small cell lung cancer (NSCLC) invasiveness and metastatic spread, remain obscure.

**Methods:**

Genomic and proteomic database analyses were performed to evaluate the potential clinical significance of MSI2 in NSCLC tumor and stromal clinical specimens. Molecular approaches were used to modify MSI2 in CAFs and determine its functional role in NSCLC cell motility in vitro using 2D and 3D models, and in metastasis in a xenograft mouse model using live-cell imaging.

**Results:**

MSI2, both gene and protein, is upregulated in NSCLC tissues and is associated with poor prognosis and high metastatic risk in patients. Interestingly, MSI2 is also upregulated in NSCLC stroma and activated fibroblasts, including CAFs. Depletion of MSI2 in CAFs by CRISPR-Cas9 strongly inhibits NSCLC cell migration and invasion in vitro, and attenuates local and distant metastatic spread of NSCLC cells in vivo. The crosstalk between CAFs and NSCLC cells occurs via paracrine signaling, which is regulated by MSI2 in CAFs via IL-6. The secreted IL-6 promotes epithelial-mesenchymal transition in NSCLC cells, which drives metastasis.

**Conclusion:**

Our findings reveal for the first time that MSI2 in CAFs is important in CAF-mediated NSCLC cell invasiveness and metastasis via IL-6 paracrine signaling. Therefore, targeting the MSI2/IL-6 axis in CAFs could be effective in combating NSCLC metastasis.

**Supplementary Information:**

The online version contains supplementary material available at 10.1186/s13578-023-01158-5.

## Background

Lung cancer is one of the most common cancers and the leading cause of cancer-related mortality worldwide, with a few million deaths each year [1]. Approximately 85% of lung cancers are non-small cell lung cancer (NSCLC), which is classified into lung adenocarcinoma (LUAD; ~ 50 − 60%), lung squamous cell carcinoma (LUSC; ~ 20 − 30%), large-cell carcinoma (LCC) and others (∼10 − 20%) [2]. The majority of NSCLC deaths (~ 70%) are linked to metastasis, wherein the 5-year overall survival rate of patients with advanced stage is less than 5% [3, 4], highlighting advanced NSCLC as a major therapeutic challenge with no effective treatments [5]. Therefore, a more detailed understanding of the underlying cause of NSCLC metastasis, which is a complex series of cellular events, and the identification of potential molecular targets are crucial to the development of effective therapeutic strategies.

The tumor microenvironment (TME) plays a fundamental role in tumor growth, disease progression, and metastasis of numerous solid cancers [6], and is a hot spot in current cancer research. Cancer-associated fibroblasts (CAFs) are an abundant, active stromal component of the TME of NSCLC, which interact closely with the malignant cells [7]. Clinical data [8] and experimental studies have shown the roles of CAFs in NSCLC chemotherapy resistance [9], cell proliferation [10], and metastasis [11], e.g., via secreted pro-tumorigenic and metastatic cytokines, chemokines, and growth factors [12]. However, a detailed understanding of the interaction between CAFs and NSCLC cells, particularly the specific molecules that regulate functional CAFs to govern the metastatic activities of NSCLC cells, remains to be further investigated. The present study was conducted to investigate the potential role of Musashi-2 (MSI2) on CAFs in regulating the invasiveness and metastatic spread of NSCLC cells and its downstream signaling cascades.

MSI2 is an RNA-binding protein known to regulate diverse biological processes involving stem cell maintenance and the developmental processes of blood and neurons [13, 14]. The potential role of MSI2 in aggressive tumors has been suggested, attributable to its elevated expression in tumor tissues, which is often positively associated with tumor size, chemotherapy resistance, and metastasis [15]. In lung cancer, elevated MSI2 expression is associated with poor prognosis and disease progression in NSCLC patients [16]. Depletion of MSI2 in NSCLC cells significantly decreased cell invasion and metastasis via TGF-β signaling [17], and potentiated chemotherapy response to EGFR inhibitors in EGFR-mutated NSCLC cells [18]. To date, numerous studies have primarily focused on the functional role of MSI2 in tumor cells; however, its role in TME stromal cells is relatively unknown. Using bioinformatics analyses, we found that *MSI2* mRNA expression is upregulated in NSCLC stroma and in activated human fibroblasts, which share a common histopathological characteristic with CAFs [19]. This prompted us to further investigate whether MSI2 in CAFs may contribute to the aggressive phenotypes of NSCLC cells. We first revealed that MSI2 level is higher in CAFs obtained from NSCLC than its matched normal fibroblasts (NFs). Herein, we tested: (i) whether MSI2 in CAFs promotes NSCLC cell motility and metastasis; (ii) how CAFs regulate NSCLC cell aggressiveness, e.g., through the secretome or direct interaction; and (iii) what are key mediators that contribute to their aggressiveness.

## Methods

Full details of key resources and experimental procedures can be found in Additional file 1: Supplementary Methods.

### Bioinformatic analysis

Lung cancer survival data were analyzed using Kaplan–Meier plotter (http://kmplot.com/analysis/) according to the mRNA expression of *MSI2* (probe set: 1552364_s_at) using median to define a cut-off [20]. *MSI2* frequencies according to the expression trend were assessed using BioXpress database (https://hive.biochemistry.gwu.edu/bioxpress), which defines over- and under-expression based on ∣log_2_ fold change∣ > 0 [21] and CPTAC proteomic profiles were assessed using UALCAN (https://ualcan.path.uab.edu/analysis-prot.html) [22]. For other analyses, gene expression datasets, including NSCLC (GSE19188, GSE31552, GSE31210, GSE118370, and GSE115002), tumor, node, metastasis (TNM) staging (GSE30219), idiopathic pulmonary fibrosis and/or usual interstitial pneumonia (IPF/UIP; GSE32537 and GSE10667), and NSCLC stroma (GSE22874) were downloaded from Gene Expression Omnibus (GEO) repository (https://www.ncbi.nlm.nih.gov/gds) and re-analyzed using GEO2R (https://www.ncbi.nlm.nih.gov/geo/geo2r/) (see also Additional file 1: Table S2).

### Cell culture

Human NSCLC cell lines, including the National Cancer Institute (NCI)-H292 and NCI-H460 cells, were purchased from American Type Culture Collection (ATCC; Manassas, VA, USA). Patient-derived CAFs and its matched NFs were kind gifts from the Weizmann Institute of Science (Rehovot, Israel) [23]. Briefly, CAFs and NFs were isolated from noncancerous margins of a newly diagnosed lung cancer patient with a poorly differentiated (Grade 3) adenosquamous carcinoma, immortalized using telomerase reverse transcriptase (hTERT), and labeled with GFP fluorescence. Cells were cultured in RPMI 1640 medium (Corning, Corning, NY, USA) supplemented with 10% fetal bovine serum (FBS) and 100 U/mL penicillin/100 μg/mL streptomycin (Gibco, Grand Island, NY, USA) and maintained in a humidified incubator at 37 °C with 5% CO_2_.

### CRISPR-Cas9 system

All-in-one CRISPR-Cas9 lentiviral (lentiCRISPR v2) plasmid carrying SpCas9 and a guide RNA targeting *MSI2* (gMSI2) was obtained from GenScript (Piscataway, NJ, USA). The used oligonucleotide sequence of gMSI2 (guide 1) is ATCCCACTACGAAACGCTCC. Lentiviral particles were packaged in HEK293T cells (ATCC) using pCMV-dR8.2 dvpr and pCMV-VSV-G, and transduced into CAFs in the presence of 8 μg/mL hexadimethrine bromide (polybrene; Santa Cruz Biotechnology, Santa Cruz, CA, USA) for 48 h. Stable clones were selected with 2 μg/mL puromycin (Gibco) for at least 3 weeks. Depletion efficiency was checked prior to use by Western blotting.

### Collection of conditioned media (CM) from CAFs and NSCLC exposure

CAFs at a density of 1 × 10^6^ cells/10-cm culture dishes were cultured overnight, after which the cells were washed twice with RPMI 1640 medium and were further cultured for 48 h to generate the CM. Collected CM was centrifuged at 300 × g at 4 °C for 10 min, filtered through a 0.45-μm membrane syringe filter, and stored at − 80 °C until use. For NSCLC exposure studies, 1 × 10^5^ NCI-H292 or NCI-H460 cells were plated into 6-well plate, washed twice with RPMI 1640 medium the next day, and further incubated with a tested medium containing CAF CM and reduced serum RPMI 1640 (5% FBS) at a 1:1 ratio for 48 h. For IL-6 and its neutralization experiments, NSCLC cells were supplemented with 25 ng/mL recombinant IL-6 or 5 μg/mL IL-6-neutralizing antibody (or IgG control) in a 1:1 mixture of CM and reduced serum media.

### Cell migration and invasion assays

Transwell chambers with 8-μm pore membrane inserts (Corning) were used for cell migration and invasion assays. For invasion assay, the transwell inserts were coated with 0.5 mg/mL Matrigel (Corning). NSCLC cells (3 × 10^4^ cells/well) in RPMI 1640 medium (350 μL) were seeded into the transwell inserts. For co-culture experiments, NSCLC (1.5 × 10^4^) and CAF (1.5 × 10^4^) cells were mixed at a 1:1 ratio and seeded into the transwell inserts. The inserts were placed in the transwell chambers containing 700 μL of RPMI 1640 medium with 10% FBS. After 48 h, non-migrating/invading cells were removed from the inside of the transwell inserts using a humidified cotton swab and the migrating/invading cells on the underside of the transwell inserts were stained with Hoechst 33342. Micrographs were taken under a fluorescence microscope and cell numbers were quantified using ImageJ software.

### 3D invasion assay

A 3D invasion assay was performed as previously described [24, 25]. Briefly, a total of 5 × 10^4^ cells/mL were cultured by a hanging drop in a 1:1 mixture of CM and reduced serum RPMI 1640 (5% FBS) in the presence or absence of IL-6, IgG, or IL-6-neutralizing antibody for 72 h. Then, rounded border spheroids were collected and embedded in 1:1 Matrigel and rat type I collagen. Next, the mixture was dropped into 24-well plates and placed at 37 °C for 30 min. Culture medium containing 20 ng/mL epidermal growth factor (EGF) and 20 ng/mL basic fibroblast growth factor (FGF) was added for cytokine-induced invasion. After 72 h, 3D spheroids were visualized under an inverted phase-contrast microscope, where the invasive protrusions from the spheroids were observed. Invasive distance, which was the longest distance of protrusions projecting from each spheroid, was measured by ImageJ software. Dissemination event, which was determined by the presence of single or cluster of disseminated cells from 3D spheroids, was also counted.

### Co-injection xenograft mouse model

All animal experiments were approved by the Institutional Animal Care and Use Committee (IACUC #1602000428_R1.1) and were performed in accordance with the Guidelines for Animal Experiments at West Virginia University. Five- to six-week-old homozygous NU/J nude mice (male) were purchased from Jackson Laboratory (Bar Harbor, ME, USA) and housed under conventional barrier protection. NSCLC NCI-H292 cells were labeled with the UBC-RFP-T2A-luciferase dual reporter for live-cell tracking. Luciferase-labeled NCI-H292 cells (2 × 10^6^ cells) were mixed with Ctrl or gMSI2 CAFs (2 × 10^6^ cells) at a 1:1 ratio in 25% Matrigel in RPMI 1640 and subcutaneously implanted into both flanks of mice. Mice were weighed, and tumor growth was monitored using a vernier caliper every week. Tumor volumes (mm^3^) were calculated using the formula 0.5 × (length × width^2^). For bioluminescence imaging, mice were intraperitoneally injected with 150 mg/kg D-luciferin and monitored on an IVIS Lumina II imaging system (PerkinElmer, Waltham, MA, USA). At the end of the experiments, mice were euthanized, and primary tumors were dissected, weighed, and photographed. The lungs and other organs of mice were collected and immediately assessed for metastatic lesions using PerkinElmer’s ex vivo imaging protocol. Isolated lung tissues were formalin-fixed, paraffin-embedded, cut into 5-μm sections, and stained with hematoxylin and eosin (H&E) for histological assessment, i.e., tumor burden and metastatic foci. In the present study, mice were sacrificed at 3 weeks post inoculation when the size of primary tumors reached the maximum tumor burden permitted at 2,000 mm^3^.

### Statistical analysis

All data analyses were performed using GraphPad Prism software. An unpaired, two-tailed Student’s *t*-test or Mann–Whitney U test was used for two-group comparisons. One-way ANOVA with Tukey’s multiple comparison test was used for multiple comparisons. The data represent mean ± SD or SEM from three or more independent experiments. Chi-square test was utilized to evaluate the difference in incidence of events between control and tested groups in mice. Statistical significance is denoted at the *P* < 0.05.

## Results

### MSI2 expression is increased in NSCLC patients and associated with poor clinical outcomes

**Fig. 1:**
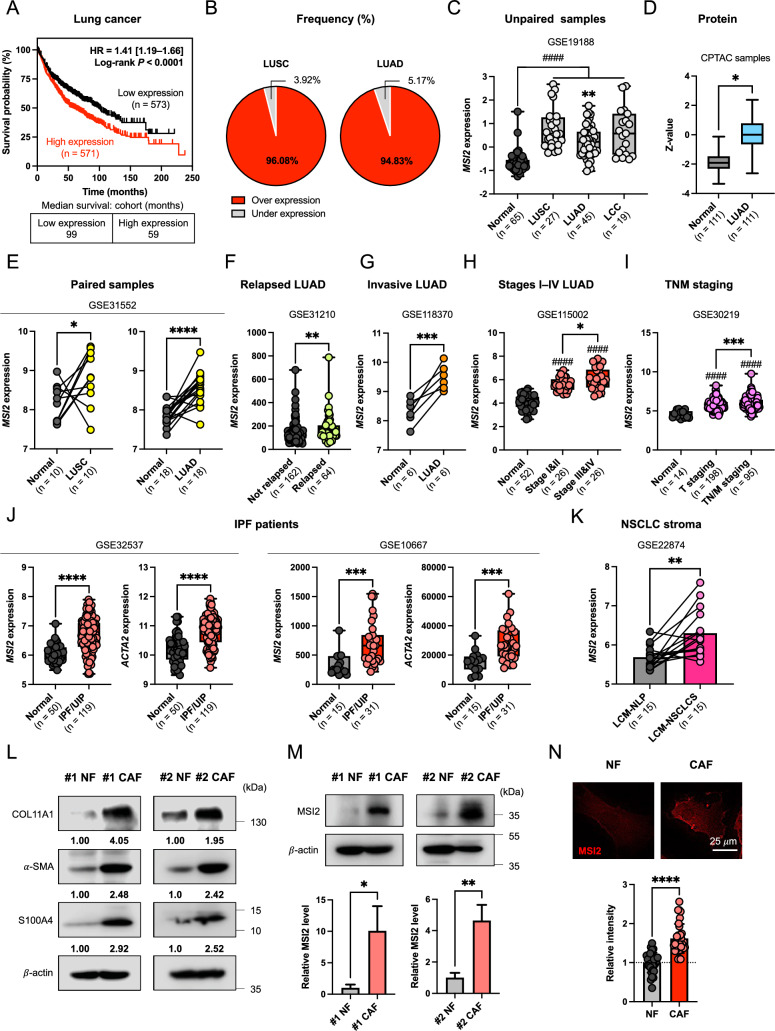
MSI2 is upregulated in NSCLC and CAFs and correlates with poor clinical outcomes in patients. **A** Kaplan–Meier survival curves of lung cancer patients according to the expression of *MSI2*. **B** The frequencies of LUSC and LUAD patients following *MSI2* expression trend (over- or under-expression) obtained from BioXpress. **C** Up-regulation of *MSI2* is observed in various NSCLC subtypes, including LUSC, LUAD, and LCC when compared with healthy lung tissue. Data are mean ± SD; ^####^*P* < 0.0001 versus normal tissues; ^**^P < 0.01 versus LUSC; one-way ANOVA with Tukey’s multiple comparison test. **D** MSI2 protein level is higher in LUAD than in normal tissues in the CPTAC database. ^*^P < 0.05 versus normal tissues; two-tailed Student’s *t*-test. **E** Comparison of *MSI2* in matched normal and tumor pairs in LUSC and LUAD patients. ^*^P < 0.05, ^****^P < 0.0001 versus normal tissues; two-tailed Student’s *t*-test. **F–I** *MSI2* expression according to aggressive clinical outcomes, including relapsed and invasive disease (F and G) and tumor staging (H and I) in the indicated lung cancer patients. Data are mean ± SD; ^*^P < 0.05, ^**^P < 0.01, ^***^P < 0.001 versus normal or not relapsed samples as indicated; ^####^*P* < 0.0001 versus normal tissues; two-tailed Student’s *t*-test or one-way ANOVA with Tukey’s multiple comparison test. **J** *MSI2* and *ACTA2* (encoding α-SMA) expression is higher in IPF/UIP lung tissues than in non-diseased tissues. Data are mean ± SD; ^***^P < 0.001, ^****^P < 0.0001 versus normal tissues; two-tailed Student’s *t*-test. **K** High *MSI2* expression in laser-capture-microdissected (LCM) NSCLC stroma (LCM-NSCLCS). ^**^P < 0.01 versus paired LCM normal lung parenchyma (LCM-NLP); two-tailed Student’s *t*-test. Notably, GEO accession numbers of the obtained RNA-seq data were indicated above the plots. **L**, **M** Western blot analysis of common CAF markers, including COL11A1, α-SMA, and S100A4 (**L**) and MSI2 (**M**), in primary (#1) and immortalized (#2) NSCLC-derived CAFs relative to its matched NFs. β-actin was used as a loading control. Quantitative analysis of MSI2 level after normalization to β-actin and relative to NFs is shown. Data are mean ± SD (n = 3); ^*^P < 0.05, ^**^P < 0.01 versus NFs; two-tailed Student’s *t*-test. **N** Representative immunofluorescence images showing MSI2 expression in CAFs and NFs under the same instrumental setting. Scale bar = 25 μm. Fluorescence intensity of MSI2 relative to NFs is shown. Data are mean ± SD (n ≥ 30 cells per group); ^****^P < 0.0001 versus NFs; two-tailed Student’s *t*-test

We first analyzed the correlation of *MSI2* expression with lung cancer survival using Kaplan–Meier plotter (n = 2166). Consistent with the previously published data obtained from a small number of NSCLC patients (n = 40) [16], patients with a high *MSI2* have a shorter overall survival than those with low *MSI2* (hazard ratio [HR] = 1.41, *P* < 0.0001) (Fig. 1A), thus supporting the prognosis value of MSI2 in NSCLC. Next, RNA sequencing (RNA-seq) data for *MSI2* were extracted from the BioXpress and GEO databases. Figure 1B and C shows that approximately 96% of LUSC and 95% of LUAD NSCLC patients have an abundant *MSI2* and that its expression is markedly higher in samples collected from patients of all NSCLC subtypes, including LUSC, LUAD, and LCC, than in healthy lung tissues (GSE19188), in agreement with the protein expression analysis using CPTAC database (Fig. 1D). To further substantiate the increased MSI2 gene and protein in NSCLC patients, we analyzed and confirmed the high *MSI2* expression in matched tumor and normal tissues obtained from LUSC and LUAD patients (GSE31552) (Fig. 1E). We also revealed the association between *MSI2* expression and aggressive clinical outcomes in three independent sets of LUAD patient cohorts (GSE31210, GSE118370, and GSE115002) and one in mixed lung cancer cohort (GSE30219). In LUAD, high *MSI2* was observed in patients who developed relapsed and invasive disease as well as those who had high-grade (III and IV) staging (Fig. 1F–H). High *MSI2* was also correlated well with advanced NSCLC—patients with TN/M staging (T > 1/N > 0/M > 0) had a significantly higher *MSI2* than those with early-stage cancer (T staging, T1N0M0) and healthy individuals (Fig. 1I), suggesting its importance in NSCLC progression and metastasis. Together, these data strongly support MSI2 as a potential prognostic biomarker and therapeutic target for advanced NSCLC.

### MSI2 is remarkably upregulated in NSCLC stroma and CAFs

Although MSI2 has been associated with various aggressive cancer phenotypes [26], its impact on the TME has not been demonstrated. Because CAFs are a major component of the NSCLC TME [7], we investigated the functional role of MSI2 in CAFs and its impact on NSCLC progression. Certain lung diseases with excessive activated fibroblasts such as IPF/UIP have been linked to lung cancer aggressiveness and poor prognosis [27, 28], and hence we analyzed *MSI2* expression in IPF/UIP patients. Analyses of two independent human IPF/UIP datasets (GSE32537 and GSE10667) revealed that both *ACTA2* (encoding α-SMA), a common activated fibroblast marker, and *MSI2* mRNA were upregulated in IPF/UIP as compared to normal lung tissues (Fig. 1J), suggesting an increased *MSI2* in activated lung fibroblasts. Importantly, bioinformatic analysis of microdissected stroma showed that *MSI2* expression was significantly higher in NSCLC stroma than in its matched normal lung parenchyma (GSE22874) (Fig. 1K). Using the same dataset, we observed an association between the frequency of CAFs with relatively high *MSI2* and staging of NSCLC (Additional file 2: Fig. S1), suggesting the plausible role of MSI2 in CAFs in NSCLC progression. We further assessed the protein level of MSI2 in primary (#1) and immortalized (#2) CAFs obtained from NSCLC patients (NSCLC CAFs) in comparison with its matched NFs. Western blot analysis showed that MSI2 level was remarkably higher in CAFs than in NFs (Fig. 1M) in parallel with an increase in COL11A1, *α*-SMA and S100A4 levels, which were used to confirm the CAF phenotype (Fig. 1L). Immunofluorescence also demonstrated an increased abundance of MSI2 protein in the cytoplasm of CAFs (Fig. 1N; Additional file 2: Fig. S2). Together, these results indicate that MSI2 is upregulated in NSCLC stroma and CAFs.

### CAFs regulate NSCLC cell motility via MSI2

**Fig. 2:**
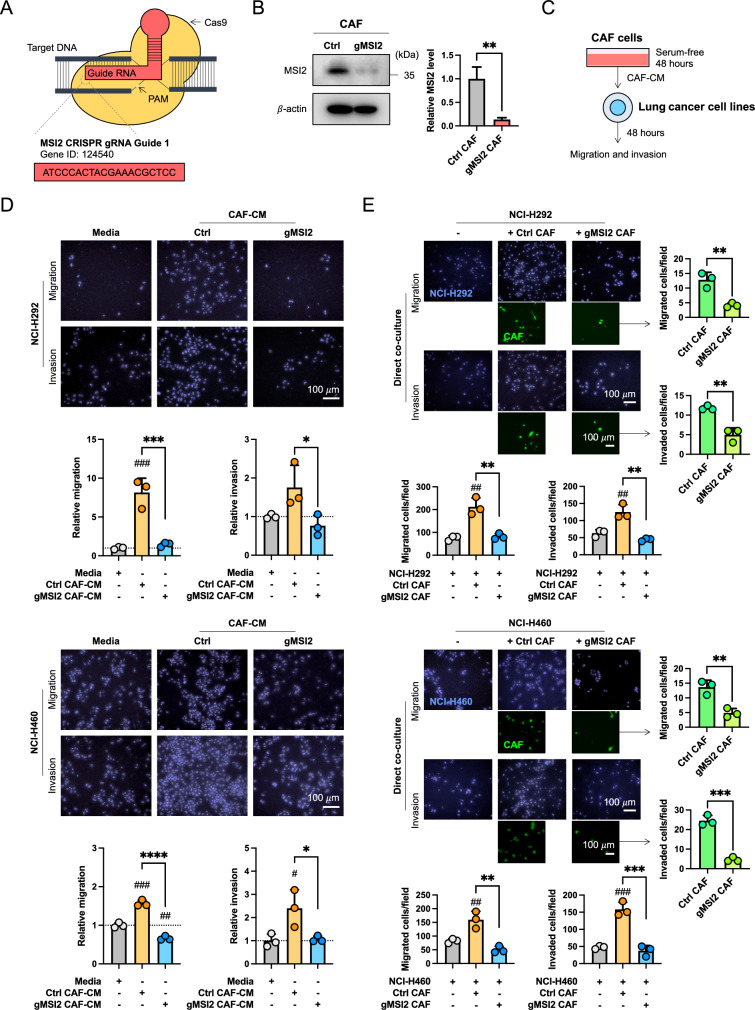
MSI2 is required for CAFs to promote NSCLC cell motility. **A** Schematic illustration of MSI2 depletion mediated by CRISPR-Cas9 system. The oligo sequences of gRNA against *MSI2* is shown. **B** CAFs were transfected with either lentiviral expressing all-in-one *MSI2* gRNA and Cas9 (gMSI2) or an empty vector (control, Ctrl). The depletion efficiency of MSI2 was measured by Western blotting (left). β-actin was used as a loading control. Quantitative analysis of MSI2 level, normalized to β-actin and relative to Ctrl CAFs (right). Data are mean ± SD (n = 3); ^**^P < 0.01 versus Ctrl CAFs; two-tailed Student’s *t*-test. **C** Workflow diagram showing the collection of CM from Ctrl and gMSI2 CAFs, following by Transwell migration and invasion assays of NSCLC cells. **D** Loss of MSI2 in CAFs inhibits NSCLC NCI-H292 (upper) and NCI-H460 (lower) migration and invasion upon exposure to CM from CAFs. Representative micrographs of migrating/invading cells stained with Hoechst 33342 are shown. Scale bar = 100 μm. Bar graph depicts relative cell migration and invasion. Data are presented as mean ± SD (n = 3); ^#^*P* < 0.05, ^##^*P* < 0.01, ^###^*P* < 0.001 versus media; ^*^P < 0.05, ^***^P < 0.001, ^****^P < 0.0001 versus Ctrl CAF-CM; one-way ANOVA with Tukey’s multiple comparison test. **E** Migration and invasion capabilities of NSCLC NCI-H292 (upper) and NCI-H460 (lower) cells upon direct co-culture with either Ctrl CAFs or gMSI2 CAFs (ratio 1:1). Representative micrographs of migrating/invading cells stained with Hoechst 33342 are shown. Scale bar = 100 μm. Migrated/invaded cells were counted and plotted. Data are mean ± SD (n = 3); ^##^*P* < 0.01 ^###^*P* < 0.001 versus NSCLC cells alone; ^**^P < 0.01, ^***^P < 0.001 versus NSCLC cells with Ctrl CAFs; one-way ANOVA with Tukey’s multiple comparison test. CAFs were GFP labeled, allowing the specific detection of migrated/invaded CAFs (right). Data are mean ± SD (n = 3); ^**^P < 0.01, ^***^P < 0.001 versus Ctrl CAFs; two-tailed Student’s *t*-test

To investigate the impact of MSI2 in CAFs on NSCLC progression, we genetically depleted MSI2 in CAFs (gMSI2 CAFs) using a lentivirus containing gRNA sequence against *MSI2* using CRISPR-Cas9 technology (Fig. 2A). Western blotting confirmed a decrease in MSI2 protein level in gMSI2 CAFs relative to empty vector control CAFs (Ctrl CAFs) (Fig. 2B). Previous studies have indicated that CAFs promote tumor progression primarily through paracrine signaling [12]; therefore, we collected CM from 48 h-culture of both Ctrl and gMSI2 CAFs to study their effects on NSCLC cell growth, colony formation, and cell invasion and migration. First, we showed that exposure of NSCLC NCI-H292 or NCI-H460 cells to gMSI2 CAF-derived CM (gMSI2 CAF-CM) for up to 5 days did not have any significant effect on cell proliferation as compared to those treated with Ctrl CAF-derived CM (Ctrl CAF-CM) (Additional file 2: Fig. S3A). Likewise, exposure of the both NSCLC cell lines to each CM did not alter their colony-forming ability (Additional file 2: Fig. S3B). Consistent with these findings, no significant changes were observed in their self-renewal ability as determined by the spheroid formation assay, except for a minor effect observed in NCI-H292 cells (Additional file 2: Fig. S3C). Hence, depletion of MSI2 in CAFs has no appreciable effect on overall NSCLC growth in vitro.

Because CAFs interact with NSCLC cells to promote metastasis [3], and activated cell motility is a hallmark of cancer [29], we investigated the importance of CAF MSI2 in NSCLC cell migration and invasion. In transwell assays, the CM from Ctrl and gMSI2 CAFs were collected from serum-free media at 48 h, after which they were exposed to NSCLC cells and their migratory and invasive properties were evaluated at 48 h, as schematically depicted in Fig. 2C. We found that exposure of NSCLC NCI-H292 and NCI-H460 cells with Ctrl CAF-CM promoted both cell migration and invasion when compared to those treated with control medium (basal RPMI 1640), thus validating that CAFs promoted NSCLC cell motility through paracrine signaling. Remarkably, exposure to gMSI2 CAF-CM led to a dramatic decrease in NSCLC cell migration and invasion to a similar degree as exposure to control medium (Fig. 2D). The inhibitory effect of gMSI2 CAF-CM on NSCLC cell migration was confirmed by a scratch wound healing assay (Additional file 2: Fig. S4). We noted that NSCLC cell proliferation was not affected by these CM during the 48-h experimental period (Additional file 2: Fig. S5), thereby substantiating that the observed inhibitory effects of gMSI2 CAF-CM on cell migration and invasion were not caused by impaired cell proliferation. To mimic the direct interaction between CAFs and NSCLC cells under real conditions, we co-cultured CAFs and NSCLC cells in the upper chamber of the Transwell system and analyzed for cell invasion and migration as previously described. We found that NSCLC NCI-H292 and NCI-H460 cells exhibited increased migratory and invasive activities only when exposed with Ctrl CAFs, but not gMSI2 CAFs, as compared to the counterpart NSCLC cells alone (Fig. 2E, left). Furthermore, gMSI2 CAFs, which are GFP labeled, showed impaired cell motility when compared to Ctrl CAFs, as measured by GFP fluorescence (Fig. 2E, right; Additional file 2: Fig. S6). Notably, the impaired cell motility was similarly observed in gMSI2 CAFs even without tumor co-culture (Additional file 2: Fig. S7), thereby ruling out the negative feedback from NSCLC cells. Together, these data indicate that MSI2 in CAFs is crucial to the migration and invasion of NSCLC cells.

### MSI2 in CAFs is critical for CAF-mediated NSCLC metastasis in vivo

**Fig. 3:**
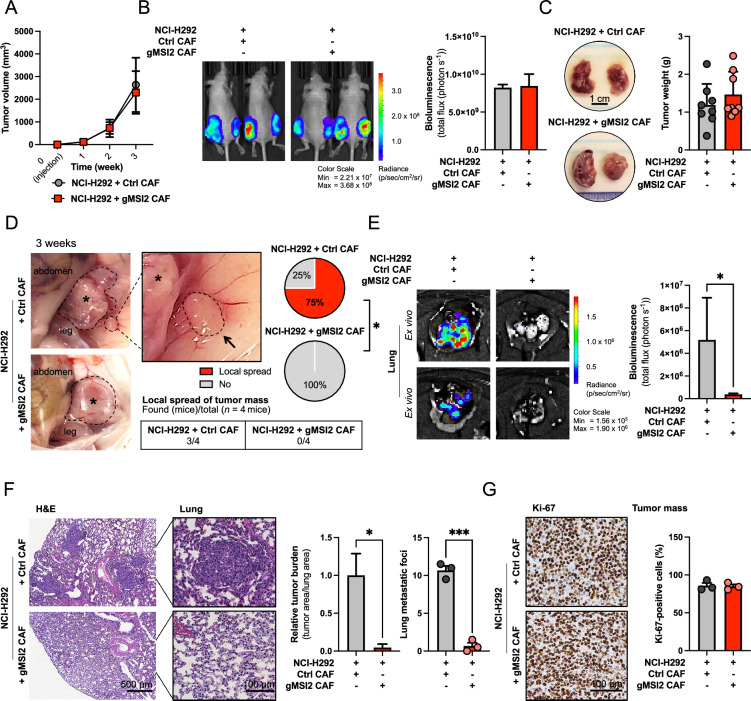
MSI2 deficiency in CAFs inhibits NSCLC metastasis in vivo. **A–C** MSI2 deficiency in CAFs has no effect on tumor growth. Luciferase-labeled NSCLC NCI-H292 cells were subcutaneously injected with Ctrl CAFs or gMSI2 CAFs (ratio 1:1) into both flanks of NU/J mice. **A** Tumor volume was monitored over three-week period. Data are mean ± SD (n = 8; 2 tumors/mice). **B** Representative bioluminescence images and its quantified bioluminescence signals at primary sites (right) at 3 weeks post inoculation. Data are mean ± SD (n = 4). **C** Representative images of gross tumors (left) and weight of resected tumors at week 3 (right). Scale bar = 1 cm. Data are mean ± SD (n = 8). **D** Representative images of tumor xenografts showing local spread of tumor mass (arrowhead). Asterisk, primary tumor mass. Frequencies of mice with or without a local spread were compared between NSCLC with Ctrl or gMSI2 CAFs. ^*^P < 0.05 versus NCI-H292 cells with Ctrl CAFs; chi-squared test. **E** Representative bioluminescence images of the dissected lungs and its quantified bioluminescence signals. Data are mean ± SEM (n = 4); ^*^P < 0.05 versus NCI-H292 cells with Ctrl CAFs; Mann–Whitney U test, two-sided. **F** Representative micrographs of H&E-stained lung tissues. Scale bars = 500 μm or 100 μm. Relative lung metastasis area and lung metastatic foci were quantified from the H&E micrographs using ImageJ. Data are mean ± SD (n = 3); ^*^P < 0.05, ^***^P < 0.001 versus NCI-H292 cells with Ctrl CAFs; two-tailed Student’s *t*-test. **G** Representative IHC micrographs of primary tumors stained for Ki-67. Scale bar = 100 μm. Percentage of Ki-67-positive cells relative to total number of cells was assessed. Data are mean ± SD (n = 3)

Based on the observations that MSI2 is upregulated in clinical NSCLC stroma and CAFs, and that MSI2 in CAFs regulates the migratory and invasive capabilities of NSCLC cells in vitro, we next tested the effect of CAF MSI2 on NSCLC metastasis in vivo using a co-injection xenograft mouse model [10, 30, 31]. Stable luciferase-expressing NSCLC NCI-H292 cells were mixed with Ctrl or gMSI2 CAFs at a 1:1 ratio and subcutaneously co-implanted into the bilateral flanks of NU/J mice. Tumor development was evaluated weekly using IVIS live-cell imaging (see also Additional file 2: Fig. S8A, B). Throughout the study, no significant change in the body weight of mice was observed between the two groups that received NSCLC NCI-H292 cells with Ctrl CAFs or gMSI2 CAFs (Additional file 2: Fig. S8C). The two mouse groups also showed no significant difference in primary tumor size (Fig. 3A), indicating that CAF MSI2 had no remarkable effect on tumor growth. These results are consistent with the luciferase bioluminescence signals (Fig. 3B) and with the direct tumor weight measurements post-mortem (Fig. 3C). Together, these results strongly indicate that MSI2 has no influence on NSCLC tumor growth in vivo, in agreement with the in vitro data on NSCLC cell growth. To evaluate the potential role of CAF MSI2 in NSCLC metastasis, mice were sacrificed at week 3 after inoculation and lung tissues were collected for ex vivo bioluminescence and tumor burden analysis on histological sections. We observed local metastatic spread from primary tumor in approximately 75% of cases in the control group receiving NCI-H292 cells and Ctrl CAFs, but this was not observed in the mouse receiving NCI-H292 cells and gMSI2 CAFs (Fig. 3D). Moreover, ex vivo bioluminescence analysis showed a significant decrease in tumor signals in the lungs of gMSI2 CAF group, indicating that MSI2-deficient CAFs inhibited NSCLC lung metastasis (Fig. 3E). Decrease in tumor signals in organs other than the lungs, i.e., brain, kidney, and spleen, was also observed in the gMSI2 CAF group, but there was no statistically significant difference in signals when compared to Ctrl CAF group (Additional file 2: Fig. S8D). Finally, H&E staining results confirmed that the Ctrl CAF group had a considerably higher lung metastatic burden and lung metastatic foci than the gMSI2 CAF group (Fig. 3F). Notably, immunohistochemistry (IHC) analysis revealed no change in Ki-67 staining in primary tumor mass in the two groups, thus confirming that CAF MSI2 does not play a significant role in NSCLC tumor growth (Fig. 3G). Taken together, both in vitro and in vivo studies strongly indicate the role of CAF MSI2 in promoting NSCLC metastasis without impacting tumor cell growth.

### MSI2-deficient CAFs have impaired IL-6 secretion

**Fig. 4:**
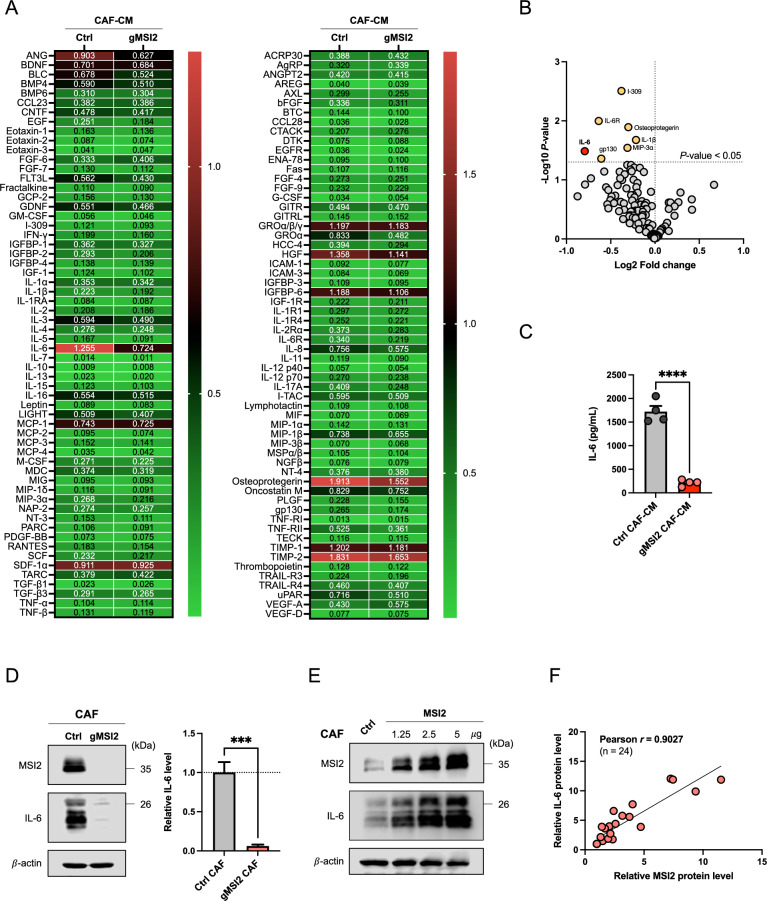
CAF MSI2 deficiency decreases IL-6 secretion. **A** The presence of tumorigenic cytokines in the CM from Ctrl CAFs and gMSI2 CAFs was analyzed using a multiplex human cytokine array. Quantification of the cytokine profile was performed by densitometry using ImageJ software. Data are normalized to the positive control (n = 4; two biological replicates), averaged, and represented in a heatmap. See also Additional file 2: Fig. S9 for cytokine array dot blots. **B** Volcano plot of human cytokine antibody array in (A) shows differential expression of several soluble factors between Ctrl CAF-CM and gMSI2 CAF-CM. It is worth noting that IL-6 decreases the most upon depletion of MSI2 in CAFs (red dot). *P* < 0.05 are in yellow dots; two-tailed Student’s *t*-test. **C** IL-6 ELISA assessing and confirming lower IL-6 levels in gMSI2 CAF-CM versus Ctrl CAF-CM. Data are mean ± SD (n = 4); ^****^P < 0.0001 versus Ctrl CAF-CM; two-tailed Student’s *t*-test. **D** Western blot analysis of IL-6 in gMSI2 CAFs in comparison to Ctrl CAFs. β-actin was used as a loading control. Data are mean ± SD (n = 3); ^***^P < 0.001 versus Ctrl CAFs; two-tailed Student’s t-test. **E** Western blot analysis of IL-6 upon MSI2 overexpression with increasing amounts of plasmid (0 − 5 μg). Ctrl CAFs were transfected with pcDNA empty vector. **F** Correlation analysis of the protein levels of MSI2 and IL-6 in CAFs

Having demonstrated that a crosstalk between NSCLC-derived CAFs and NSCLC cells occurs primarily via paracrine signaling, we further identified potential cytokines involved in the observed metastatic effect. A multiplex human cytokine antibody array was used to detect various soluble factors secreted from the Ctrl and gMSI2 CAFs into the CM (Fig. 4A; Additional file 2: Fig. S9**)**. As shown in Fig. 4B, depletion of MSI2 in CAFs affected their secretome—lower secretion of several soluble factors known to be associated with metastasis and poor prognosis in NSCLC patients, such as osteoprotegerin [32], MIP-3α [33], and IL-1β [34], was detected. Remarkably, we observed that a family of IL-6 signaling factors, including IL-6, IL-6R, and gp130, were markedly decreased in the gMSI2 CAF-CM compared to those of Ctrl CAF-CM. Given that IL-6 was the most significantly downregulated cytokine in gMSI2 CAF-CM (Fig. 4B), and that it is known to be associated with poor survival in NSCLC patients [35] and increased cell motility [36], we further investigated whether IL-6 is a key mediator of CAF-induced NSCLC cell invasion. Using quantitative ELISA, we showed that secreted IL-6 in the gMSI2 CAF-CM was substantially lower than that in the Ctrl CAF-CM (Fig. 4C), thus supporting IL-6 as a prime candidate in CAF regulation of NSCLC progression.

To confirm that IL-6 is a downstream target of MSI2 in CAFs, we first compared the IL-6 level in gMSI2 CAFs and Ctrl CAFs using Western blotting. Consistent with the results obtained from CM by cytokine antibody array and ELISA, gMSI2 CAFs had remarkably lower IL-6 level than Ctrl CAFs (Fig. 4D). Next, Ctrl CAFs were transfected with various doses (0–5 μg) of MSI2 transgene, and MSI2 and IL-6 levels were evaluated by Western blotting. Figure 4E shows that an increasing amount of MSI2 transgene in CAFs caused an increase in IL-6 in a dose-dependent manner. A correlation plot of MSI2 and IL-6 reveals their positive correlation with a correlation coefficient (*r*) of 0.9027 (Fig. 4F). In agreement with this, IHC analysis documented overall lower IL-6 in primary tumors obtained from mice co-injected with NCI-H292 cells and gMSI2 CAFs when compared to those with Ctrl CAFs (Additional file 2: Fig. S10). Previous studies reported that IL-6 in CAFs was activated via JAK2/STAT3 and NF-κB signaling pathways [37–39]. Here, we observed a tremendous downregulation of *JAK2*, *STAT3*, *NFKB1*, *NFKB2*, *P65*, and *REL* in gMSI2 CAFs when compared to Ctrl CAFs in parallel with a downregulation of *IL6* (Additional file 2: Fig. S11). Altogether, these results indicate that MSI2 regulates IL-6 in CAFs likely via JAK2/STAT3 and NF-κB signaling pathways.

### CAFs promote NSCLC cell motility via MSI2-mediated IL-6 secretion

**Fig. 5:**
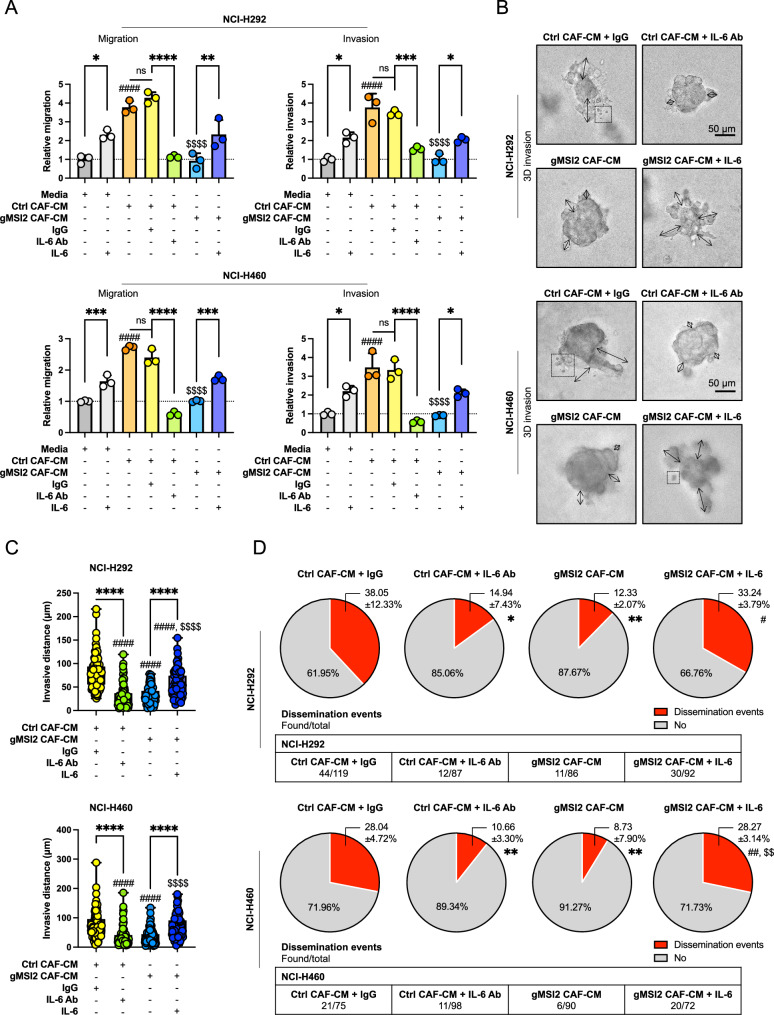
MSI2-driven IL-6 secretion in CAFs regulates NSCLC cell migration and invasion. **A** Transwell (2D) assay was performed to evaluate NSCLC cell migration and invasion. NSCLC NCI-H292 (upper) and NCI-H460 (lower) cells were cultured in media or Ctrl CAF-CM and gMSI2 CAF-CM supplemented with recombinant IL-6, control IgG Ab, or neutralizing anti-IL-6 Ab, as indicated. See also Additional file 2: Fig. S12 for representative micrographs of migrating/invading cells. Bar graph depicts relative cell migration and invasion. Data are mean ± SD (n = 3); ^####^*P* < 0.0001 versus media; ^$$$$^*P* < 0.0001 versus Ctrl CAF-CM; ^*^P < 0.05, ^**^P < 0.01, ^***^P < 0.001, ^****^P < 0.0001 versus indicated group; one-way ANOVA with Tukey’s multiple comparison test. ns, not significant. Ab, antibody. **B** CAF MSI2 promotes NSCLC cell motility through IL-6 in a 3D invasion assay. NSCLC NCI-H292 (upper) and NCI-H460 (lower) cells were processed to spheroids in either Ctrl CAF-CM or gMSI2 CAF-CM supplemented with control IgG Ab, neutralizing anti-IL-6 Ab, or recombinant IL-6, as indicated. Spheroids were embedded in extracellular matrix for 72 h, and invasion and dissemination events were evaluated. Representative micrographs of 3D spheroids are shown; arrows indicate invasive distance projecting from the spheroids; boxes indicate dissemination events. Scale bar = 50 μm. **C** Plots showing the longest invasive distance measured from a spheroid. Data are mean ± SD (n > 85 spheroids per group; four biological replicates); ^####^*P* < 0.0001 versus Ctrl CAF-CM; ^$$$$^*P* < 0.0001 versus Ctrl CAF-CM + IL-6 Ab; ^****^P < 0.0001 versus indicated group; one-way ANOVA with Tukey’s multiple comparison test. **D** Frequency of dissemination events are shown as a pie chart for better visualization. Data are mean ± SD (n = 3 or 4); ^*^P < 0.05, ^**^P < 0.01 versus Ctrl CAF-CM with IgG; ^#^*P* < 0.05, ^##^*P* < 0.01 versus gMSI2 CAF-CM; ^$$^*P* < 0.01 versus Ctrl CAF-CM with IL-6 Ab; one-way ANOVA with Tukey’s multiple comparison test. Number of total events were also shown

To further investigate the potential role of CAF-secreted IL-6 in the regulation of NSCLC cell motility, we conducted a series of experiments. First, we tested whether IL-6 could have a direct effect on NSCLC cell motility in the tested system. NSCLC NCI-H292 and NCI-H460 cells were treated with recombinant human IL-6 or neutralizing antibody (IL-6 Ab), and their effects on cell migration and invasion were examined using Transwell assays (Fig. 5A; Additional file 2: Fig. S12). Compared to control (basal media) treatment, IL-6 treatment significantly increased the migration and invasion of NSCLC cells (Fig. 5A; media versus media + IL-6). Blocking of IL-6 with IL-6 Ab in the Ctrl CAF-CM completely reversed the promoting effect of Ctrl CAF-CM on NSCLC cell motility (Fig. 5A; Ctrl CAF-CM+/−IgG or IL-6 Ab) to a similar degree as that observed in NSCLC cells treated with gMSI2 CAF-CM. Collectively, these data suggest that secreted IL-6 from CAFs promotes the migratory and invasive activities of NSCLC cells.

Having demonstrated that MSI2-deficient CAFs have limited capacities to promote NSCLC cell motility and to secrete IL-6, we postulated that MSI2 in CAFs may promote NSCLC cell motility via IL-6. Hence, the addition of IL-6 to gMSI2 CAF-CM should be able to rescue the inhibitory effect of MSI2 depletion in CAFs on NSCLC cell motility. Indeed, our result demonstrated such rescue effect (Fig. 5A; gMSI2 CAF-CM+/−IL-6). It should be noted that the indicated treatments had no effect on cell proliferation as determined by quantitative cell count and CCK-8 assays (Additional file 2: Fig. S13A, B). To further demonstrate that IL-6 is a downstream effector of MSI2 in CAFs, we performed a 3D invasion assay that closely mimicked the in vivo milieu [40]. Figure 5B shows that NSCLC NCI-H292 and NCI-H460 tumor spheroids incubated with Ctrl CAF-CM exhibited increased invasive protrusions into the surrounding matrix, whereas NSCLC spheroids incubated with gMSI2 CAF-CM showed the opposite effect. Quantitative analysis of the invasive distance from NSCLC spheroids of different groups is shown in Fig. 5C. Importantly, the occurrence of disseminated events was significantly reduced in NSCLC spheroids exposed to Ctrl CAF-CM in the presence of IL-6 Ab (Fig. 5D; Ctrl CAF-CM+/−IgG or IL-6 Ab), and by contrast the reduced disseminated events in NSCLC spheroids exposing to gMSI2 CAF-CM were rescued by the presence of IL-6 (Fig. 5D; gMSI2 CAF-CM+/−IL-6). Together, these findings strongly support that MSI2 in CAFs regulates NSCLC cell migration, invasion, and metastatic spread via paracrine IL-6 secretion.

### MSI2 deficiency in CAFs attenuates epithelial-mesenchymal transition (EMT) in NSCLC cells

**Fig. 6:**
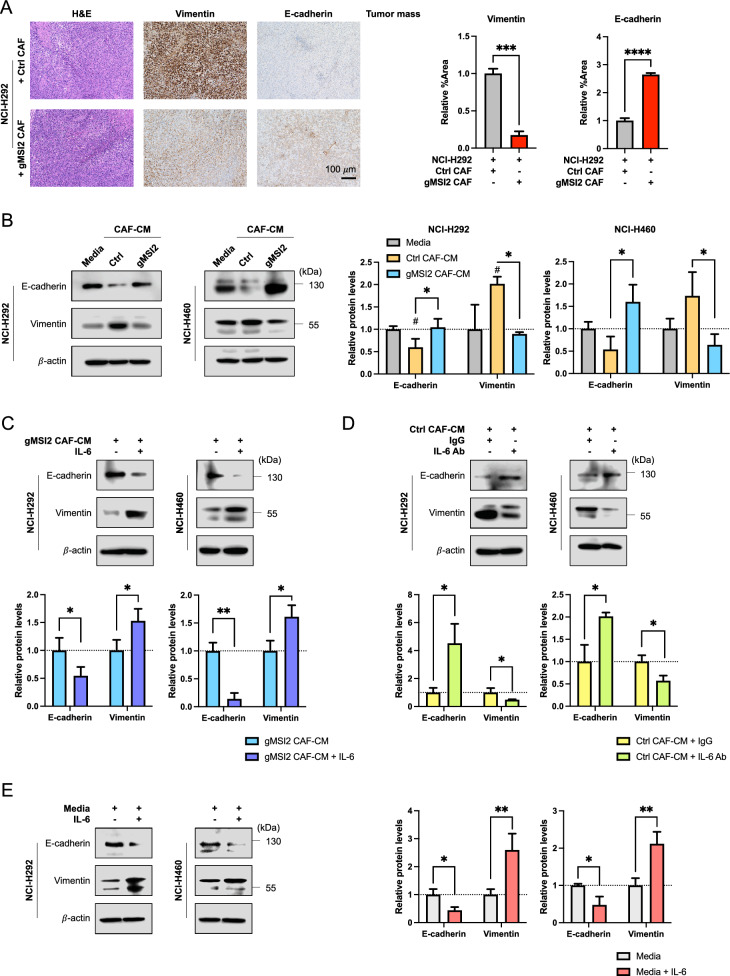
MSI2 deficiency in CAFs inhibits NSCLC EMT and metastasis via IL-6. **A** H&E staining and IHC analysis of EMT-related markers were performed on primary tumors collected from mouse xenografts. Representative micrographs and quantitative analysis of mesenchymal marker vimentin and epithelial marker E-cadherin using ImageJ software are shown. Scale bar = 100 μm. Data are mean ± SD (n = 3); ^***^P < 0.001, ^****^P < 0.0001 versus NCI-H292 cells with Ctrl CAFs; two-tailed Student’s *t*-test. **B** CAF MSI2 mediates EMT in NSCLC cells. Western analysis of E-cadherin and vimentin in NSCLC NCI-H292 (left) and NCI-H460 (right) cells after exposure to Ctrl CAF-CM or gMSI2 CAF-CM for 48 h (see also Additional file 2: Fig. S14 for more EMT markers). β-actin was used as a loading control. Data are mean ± SD (n = 3); ^#^*P* < 0.05 versus media; ^*^P < 0.05 versus Ctrl CAF-CM; one-way ANOVA with Tukey’s multiple comparison test. **C** Inhibition of EMT in NSCLCs by MSI2-depleted CAF-CM is rescued by an addition of recombinant IL-6. Western analysis of E-cadherin and vimentin in NSCLC NCI-H292 (left) and NCI-H460 (right) cells after exposure to gMSI2 CAF-CM with or without recombinant IL-6 for 48 h. β-actin was used as a loading control. Data are mean ± SD (n = 3); ^*^P < 0.05, ^**^P < 0.01 versus untreated; two-tailed Student’s *t*-test. **D** Neutralization of IL-6 reverses CAF-CM-induced EMT in NSCLC cells. Western analysis of E-cadherin and vimentin in NSCLC NCI-H292 (left) and NCI-H460 (right) cells after exposure to Ctrl CAF-CM in the presence of anti-IL6 neutralizing antibody (IL-6 Ab) or isotype-matched IgG control (IgG). β-actin was used as a loading control. Data are mean ± SD (n = 3); ^*^P < 0.05 versus IgG control; two-tailed Student’s *t*-test. **E** IL-6 activates EMT in NSCLC cells. Western analysis of E-cadherin and vimentin in NSCLC NCI-H292 (left) and NCI-H460 (right) cells upon direct exposure to recombinant IL-6 for 48 h (see also Additional file 2: Fig. S15 for more EMT markers). β-actin was used as a loading control. Data are mean ± SD (n = 3 or 4); ^*^P < 0.05, ^**^P < 0.01 versus media; two-tailed Student’s *t*-test

**Fig. 7:**
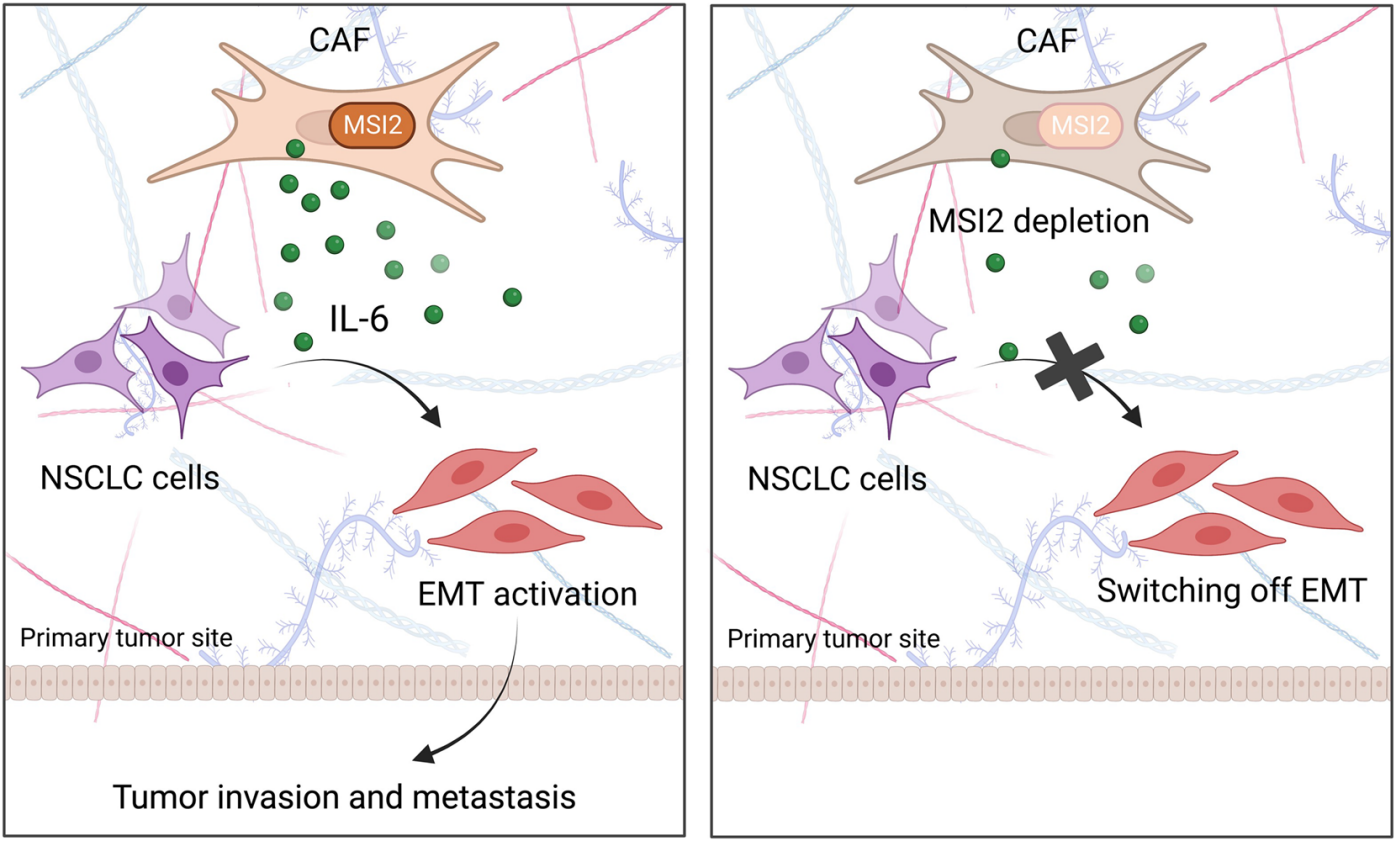
Graphical illustration highlighting our findings that CAFs promote NSCLC invasion and metastasis via MSI2-driven IL-6 secretion. MSI2 is upregulated in CAFs, resulting in IL-6 secretion, which mediates the tumor promoting effect of CAFs via the activation of EMT in NSCLC cells, resulting in tumor metastasis. By contrast, depletion of MSI2 in CAFs inhibited paracrine IL-6, which limits EMT in NSCLC cells, thus preventing NSCLC spreading

We next addressed how IL-6 regulates NSCLC cell motility. We postulated that CAF-secreted IL-6 may regulate cell motility via EMT since EMT is essential for tumor invasion and metastasis [41]. Indeed, we observed in the in vivo co-injection experiments that NSCLC NCI-H292 cells implanted with gMSI2 CAFs, but not with Ctrl CAFs, expressed a high level of the epithelial marker E-cadherin and a low level of the mesenchymal marker vimentin in the primary NSCLC tumor tissues, as evaluated by IHC (Fig. 6A), indicating an impaired EMT in the gMSI2 CAF group. To confirm the effect of CAF MSI2 on EMT in NSCLC cells, Western blotting was performed on NSCLC NCL-H292 and NCI-H460 cells upon exposure to CM from gMSI2 CAFs or Ctrl CAFs. As expected, NSCLC cells treated with Ctrl CAF-CM exhibited a high expression of vimentin and low expression of E-cadherin relative to the medium control, consistent with the previous finding showing the ability of CAFs to promote EMT [42]. Importantly, we found that MSI2 depletion in CAFs led to a repression of EMT in NSCLC cells—our data revealed a low level of mesenchymal markers (vimentin, N-cadherin, and Slug) and a high level of epithelial markers (E-cadherin and ZO-1) in the gMSI2 CAF-CM group as compared with Ctrl CAF-CM (Fig. 6B; Additional file 2: Fig. S14). Such repressive effect can be rescued by the addition of IL-6 to the CM collected from gMSI2 CAFs, suggesting that CAF MSI2 regulates EMT in NSCLC cells via IL-6 signaling (Fig. 6C; Additional file 2: Fig. S14). Additionally, neutralizing antibody experiments showed that the mesenchymal marker vimentin was abundant in NSCLC cells treated with control IgG antibody, whereas it was downregulated in the cells treated with IL-6 Ab. Opposite results were observed with the epithelial marker E-cadherin in these cells (Fig. 6D). To validate the link between IL-6 and EMT in NSCLC cells, NCI-H292 and NCI-H460 cells were directly treated with recombinant human IL-6, and EMT markers were evaluated by Western blotting. Figure 6E shows the activation of EMT in NSCLC cells upon IL-6 treatment (see also Additional file 2: Fig. S15 for additional proteins). Together, these results support the crucial role of MSI2 in CAFs in promoting EMT in NSCLC cells via IL-6 secretion. Our results also revealed the novel regulatory axis of the CAF MSI2/IL-6−NSCLC EMT as a key driver of metastasis in NSCLC cells (Fig. 7).

## Discussion

The TME stroma actively participates in tumor metastasis of various cancers, including NSCLC. In this study, we found that CAFs derived from NSCLCs mediate the motility and metastatic spread of NSCLC cells without affecting their growth and proliferation, supporting the concept that CAFs represent an attractive target for advanced NSCLC. We further unveiled a novel role of the RNA-binding protein MSI2 in regulating CAF interactions with NSCLC cells, which occur via paracrine signaling. The clinical significance of MSI2 in NSCLC is strongly supported by genomic and proteomic database analyses in NSCLC tissues and stroma, which showed the association of MSI2 expression with the disease as well as with its progression and poor prognosis in NSCLC patients.

MSI2 is extensively expressed in both solid and hematological malignancies [13, 15]. Although the high level of MSI2 has been linked to high-grade NSCLC and metastatic staging in patients [16, 17], which was validated in the present study (Fig. 1A–I), the expression of MSI2 and its functional role in TME stromal cells during tumor progression and metastasis have never been studied. Regarding the role of MSI2 in activated fibroblasts, we observed a correlation between *MSI2* and *ACTA2* (encoded α-SMA) expression in IPF/UIP tissues (Additional file 2: Fig. S16), suggesting that high MSI2 is a frequent phenomenon in activated fibroblasts. Given that CAFs are activated fibroblasts that traditionally express high α-SMA, we detected and revealed an up-regulation of MSI2 in NSCLC-derived CAFs (Fig. 1L–N), which could be of clinical significance due to its detectable abundance in the NSCLC stroma (Fig. 1K). Our data indicated for the first time that MSI2 in CAFs plays an important role in promoting NSCLC cell migration and invasion, which are initial steps in metastasis [43]. Depletion of MSI2 in CAFs not only impaired its own cell motility (Fig. 2E; Additional file 2: Fig. S7) but also inhibited NSCLC cell motility, in part, through a paracrine signaling (Fig. 2D). Collected CM from MSI2-expressed CAF culture effectively promoted NSCLC cell migration and invasion, the effects of which were abolished when using CM from MSI2-depleted CAFs. In agreement with the in vitro data, co-injection of NSCLC cells with MSI2-depleted CAFs in a xenograft mouse model resulted in decreased local and distant metastatic spreads of NSCLC cells as compared to co-injection with control CAFs (Fig. 3D–F) without affecting primary tumor growth (Fig. 3A–C, G; Additional file 2: Fig. S8). While previous studies have shown that CAFs have a significant effect on tumor cell survival/proliferation [10], we did not observe such effect, which is likely depends on the cellular context, specific cell type, and heterogeneity of the cells [44, 45]. Earlier, Qu et al. reported that conditional deletion of *Msi2* in mouse myofibroblasts in an *Msi2*^F/F^*Col1a1*-Cre mouse model attenuated hepatocellular carcinoma tumor progression [46], thus strengthening our findings that MSI2 in stromal cells is a promising target for cancer therapy. Currently, a small-molecule inhibitor of MSI2, Ro 08-2750, is undergoing a pre-clinical study [47] and has shown high in vivo efficacy against chronic lymphocytic leukemia (CLL) [48], with more similar drugs are being tested [49, 50]. As depletion of MSI2 in human NSCLC cell lines reduced its invasion and metastatic potential [17], we suggest that MSI2 small molecule drugs in NSCLC may have a dual effect, i.e., not only by targeting and weakening tumor cells but also inactivating CAFs in the TME, thereby enhancing their effectiveness in NSCLC treatment.

Multiple lines of evidence support the crosstalk between CAFs and tumor cells via paracrine signaling through the CAF secretome, leading to increased cell invasion and metastasis in various cancers [12]. Our current work supports such findings and further demonstrates the regulatory mechanisms of tumor metastasis by CAFs, which occur via the CAF MSI2/IL-6−NSCLC EMT axis. Paracrine IL-6 secretion from CAFs was identified using cytokine array and ELISA assays (Fig. 4). We further validated that secreted IL-6 is a downstream target of MSI2 in CAFs—its secretion by CAFs was strongly inhibited by MSI2 depletion. Additionally, MSI2-depleted CAFs exhibited low cellular IL-6 level (Fig. 4D), indicting the low production of IL-6 upon MSI2 depletion. Correlation analysis further revealed a positive correlation between MSI2 and IL-6 in CAFs upon MSI2 overexpression (Fig. 4E, F). IL-6 is a pleiotropic cytokine that is implicated in various cancers. For example, IL-6 inhibition by neutralizing antibody was found to reduce the metastatic potential of gastric [51], bladder [52], and lung [11] cancer cells. Consistently, NSCLC cell motility and the occurrence of in vitro disseminated events were significantly inhibited by IL-6 neutralizing antibody and conversely promoted by IL-6 (Fig. 5B–D). Clinically, IL-6 up-regulation in plasma is associated with poor outcome and advanced stage in NSCLC patients [35, 53]. Notably, we could not rule out the involvement of other cytokines and soluble factors other than IL-6, such as osteoprotegerin, MIP-3α, and IL-1β, as they were also suppressed in MSI2-depleted CAF-CM (Fig. 4A, B). However, their roles in NSCLC metastasis are beyond the scope of this study.

Given that EMT is associated with tumor metastasis [41] and that the TME can mediate EMT plasticity or reversibility [42], including those in NSCLC, we chose to investigate the EMT in NSCLC cells in response to CAFs by various means. Our ex vivo IHC data revealed that that co-injection of MSI2-positive CAFs in an NSCLC xenograft promoted EMT in NSCLC tumors, whereas MSI2-depleted CAFs lacked this ability (Fig. 6A), which was further validated in vitro (Fig. 6B–D; Additional file 2: Fig. S14). Addition of IL-6 to the CM from MSI2-depleted CAFs restored the EMT in NSCLC cells, in parallel with an observed rescue in NSCLC spreading impairment in 2D and 3D invasion assays, thus supporting secreted IL-6 as a key mediator of the tumor-promoting effects of CAFs mediated by MSI2 via EMT. The promoting role of IL-6 in EMT in NSCLC was further validated by direct IL-6 treatment (Fig. 6E; Additional file 2: Fig. S15). These results are consistent with the established role of IL-6, particularly those secreted by CAFs, in NSCLC aggressiveness [36, 54].

## Conclusion

Overall, our findings indicate the essential role of MSI2 in CAFs in regulating NSCLC metastasis and suggest this molecule as a prognostic marker and potential therapeutic target for advanced NSCLC. We revealed the novel, complicated regulatory axis of CAF MSI2/IL-6−NSCLC EMT, involving the interaction between NSCLC-derived CAFs and NSCLC cells via paracrine signaling. Our findings suggest that targeting MSI2 in advanced NSCLC patients may have a dual, synergistic effect, that is, by targeting both stromal CAFs and NSCLC cells. Our novel findings, as schematically summarized in Fig. 7, could aid in the understanding of NSCLC tumor progression and metastasis. Because of the critical role of MSI2 in NSCLC-derived CAFs, further studies might explore the involvement of MSI2 in the stroma of other tumors, which may provide the promising target for clinical utility for their advanced stage.

### Supplementary Information

**Additional file 1:** Supplementary methods. **Table S1.** Key resource table. **Table S2.** Databases used for bioinformatic analyses. **Table S3.** Primer sequences for quantitative real-time PCR (RT-qPCR).

**Additional file 2:** **Fig. S1.** *MSI2* is highly expressed in the CAFs derived from patients with advanced NSCLC. **Fig. S2.** CAFs exhibited higher α-SMA and MSI2 than NFs. **Fig. S3.** MSI2 deficiency in CAFs has no effect on NSCLC cell proliferation and clonogenic growth. **Fig. S4.** MSI2 deficiency in CAFs inhibits NSCLC cell migration. **Fig. S5.** Treatment of NSCLC cells with CM from Ctrl and gMSI2 CAFs has minimal effect on cell proliferation. **Fig. S6** MSI2 in CAFs modulates NSCLC cell motility. **Fig. S7.** MSI2 in CAFs modulates its cell motility. **Fig. S8.** MSI2 in CAFs promotes NSCLC metastasis in nude mice. **Fig. S9.** Cytokine secretion from Ctrl and gMSI2 CAFs. **Fig. S10.** IHC analysis of IL-6 in primary tumors obtained from mice bearing NCIH292 cells with Ctrl or gMSI2 CAFs. **Fig. S11.** Downregulation of multitude of genes involved in JAK2/STAT3 and NF- κB signaling pathways upon MSI2 depletion in CAFs. **Fig. S12.** IL-6 restores the inhibitory effect of MSI2-deficient CAFs on NSCLC cell migration and invasion. **Fig. S13.** Ctrl and gMSI2 CAF-CM with control IgG Ab, neutralizing anti-IL-6 Ab, or recombinant IL-6 have no appreciable effect on NSCLC cell proliferation. **Fig. S14.** MSI2 in CAFs mediates EMT activation in NSCLC via paracrine IL-6. **Fig. S15.** IL-6 activates EMT in NSCLC cells. **Fig. S16.** Expression level of *MSI2* is positively correlated with *ACTA2*.

## Data Availability

The datasets used and/or analyzed during the current study are available from the corresponding author on reasonable request. Source data are provided with this paper. Supplementary information is available in Additional file 1 and Additional file 2.

## Abbreviations

Ab: Antibody
CAF: Cancer-associated fibroblast
CM: Conditioned media
Ctrl: Control
ELISA: Enzyme-linked immunoassay
EMT: Epithelial-mesenchymal transition
GEO: Gene expression omnibus
gRNA: Guide RNA
H&E: Hematoxylin and eosin
IgG: Immunoglobulin G
IHC: Immunohistochemistry
IL-6: Interleukin-6
IPF/UIP: Idiopathic pulmonary fibrosis and/or usual interstitial pneumonia
LCC: Large-cell carcinoma
LUAD: Lung adenocarcinoma
LUSC: Lung squamous cell carcinoma
MSI2: Musashi-2
NF: Normal fibroblast
NSCLC: Non-small cell lung cancer
RNA-seq: RNA sequencing
TME: Tumor microenvironment
TNM: Tumor, node, and metastasis
α-SMA: Alpha-smooth muscle actin
