# Supplementary material for: Musashi-2 in cancer-associated fibroblasts promotes non-small cell lung cancer metastasis through paracrine IL-6-driven epithelial-mesenchymal transition
Source: Cell Biosci. 2023 Nov 8;13:205. doi: 10.1186/s13578-023-01158-5 (PMC10631049; doi:10.1186/s13578-023-01158-5)
Supplement: Supplementary file 1 — Additional file 1: Supplementary methods. Table S1. Key resource table. Table S2. Databases used for bioinformatic analyses. Table S3. Primer sequences for quantitative real-time PCR (RT-qPCR). [file 13578_2023_1158_MOESM1_ESM.pdf]

Additional file 1: Supplementary Methods

Immunofluorescence

NFs and CAFs were seeded onto glass coverslips and fixed with 4% paraformaldehyde. Next, the cells were permeabilized with 0.5% Triton X-100 in PBS, followed by blocking with 10% FBS in PBS. The cell samples were incubated with primary antibodies at 4 °C overnight, after which they were incubated with Alexa Fluor 647-conjugated secondary antibodies for 1 h at room temperature. The samples were then mounted onto a microscope slide using ProLong™ Diamond anti-fade reagent with DAPI. Fluorescence images were taken under an inverted fluorescence microscope and were analyzed using ImageJ software.

Western blotting

Following specific treatments, cells were lysed in lysis buffer containing a protease inhibitor cocktail. Then, a total protein of 30–60 μg was subjected to 7.5–12% SDS-PAGE and transferred to a PVDF membrane using wet-blot transfer apparatus. 5% non-fat milk was used as a blocking buffer. The membranes were incubated with primary antibodies overnight at 4 °C. After incubation with secondary HRP-conjugated antibodies for 2 h at room temperature, the blots were detected with ECL chemiluminescence using a digital imaging system. Band intensities were semi-quantified using ImageJ software.

Wound-healing assay

A total of 1.5×10^5 cells/well was plated into 24-well plates and incubated with culture medium. Then, a wound space was made using a sterile plastic 200-μL pipette tip, and cell debris was removed by washing with RPMI 1640 medium. CAF-CM and RPMI 1640 containing 5% FBS were mixed in a 1:1 ratio and added to the wells. Micrographs were taken

at 0 and 48 h under an inverted phase-contrast microscope. Wound spaces were measured using ImageJ software.

Cytokine antibody array

Cytokines and chemokines in CAF-CM were analyzed using the RayBiotech human cytokine antibody array C1000, according to the manufacturer's instructions. Briefly, membranes were blocked with the supplied blocking buffer, then incubated with CAF-CM overnight at 4 °C. Next, biotin-conjugated antibody and HRP-streptavidin were added and the membranes were detected using chemiluminescence.

ELISA

An ELISA kit (R&D systems) was used to quantify CAF-secreted IL-6 in CM according to the manufacturer's instructions. Briefly, ELISA plates coated with IL-6 antibody were incubated with CAF-CM containing antibody cocktail for 1 h. The plates were washed three times with washing buffer, and detection substrate and stop solution were added. Then, the signals were read at 450 nm.

Histopathology

For immunostaining of mouse xenograft tumors, mouse tissue specimens were fixed with 10% neutral buffered formalin, embedded in paraffin, and sliced sections were subjected to routine staining with hematoxylin and eosin (H&E), or immunohistochemical (IHC) staining with the indicated antibodies. All histopathological processing was performed at the West Virginia University Pathology Research Laboratories. All images were obtained by an inverted phase-contrast microscope and quantified using ImageJ software.

Proliferation assays

A total of 4 × 10^4 cells/well was seeded into 6-well plates and cultured in culture medium. On the next day, the medium was removed, and the cells were washed with RPMI 1640 medium. Then, CAF-CM and RPMI 1640 containing 5% FBS were mixed in a 1:1 ratio and added to the wells. Cells were counted at day 1, 3, and 5 after specific treatments using an automated cell counter.

Colony formation assay

A total of 500 cells/well was seeded into 6-well plates and cultured in culture medium. On the next day, the medium was removed, and the cells were washed with RPMI 1640 medium. Then, CAF-CM and RPMI 1640 containing 5% FBS were mixed in a 1:1 ratio and added to the wells. One week after plating, the cells were fixed with 4% paraformaldehyde and stained with 0.1% crystal violet. Whole well images were taken using a digital imaging system and counted by ImageJ software.

Sphere formation assay

Following specific treatments, a total of 500 cells/well was seeded into 24-well ultra-low attachment plates in MethoCult™ H4100 containing 20 ng/mL EGF, 20 ng/mL FGF, and 4 mg/mL insulin. One week after plating, micrographs were taken under an inverted phase-contrast microscope, and sphere number and size were quantified using ImageJ software.

CCK-8 assay

Following specific treatments, a total of 3 × 10^3 cells/well was seeded into 96-well plates. At 0, 24, and 48 h, a total of 10 μL/well of a CCK-8 solution was added and incubated for 4 h at

37 °C. Optical absorbance was then measured at the wavelength of 450 nm using a microplate reader and corrected for background reading.

Overexpression plasmid and transfection

CAFs were transfected with either MSI2 (Genscript) or control pcDNA3.1 plasmid using Lipofectamine 3000 reagent according to the manufacturer's instructions. At 48 h post-transfection, cells were subjected to Western analysis for MSI2 and IL-6 levels to investigate their potential correlation.

Quantitative real-time PCR (RT-qPCR)

Total RNA was extracted from Ctrl and gMSI2 CAFs by using Tri reagent, followed by quantification using a NanoDrop 2000 spectrophotometer. The generation of complementary DNA (cDNA) was performed using RevertAid First Strand cDNA Synthesis Kit according to the manufacturer's instructions. RT-qPCR was performed using the CFX384 Touch™ Real-Time PCR Detection System with SYBR™ Select Master Mix. Additional file 1: Table S3 shows the primer sequences that were used in this study. Relative expression of each gene was normalized to *GAPDH*, which served as a housekeeping gene.

Table S1. Key resource table.

Name	Source	Cat# No.
**Antibodies**		
Mouse anti-MSI2 (WB 1: 500)	Santa Cruz Biotechnology	sc-517212
Mouse anti-MSI2 (IF 1: 200)	Origen	TA506196
Mouse anti-β-actin (1: 1000)	Santa Cruz Biotechnology	sc-47778
Rabbit anti-S100A4 (1: 1000)	Abcam	ab27957
Mouse anti-α-SMA (1: 1000)	Cell Signaling Technology	48938S
Rabbit anti-COL11A1 (1: 1000)	Cell Signaling Technology	96321
Rabbit anti-E-cadherin (1: 500)	Cell Signaling Technology	3195T
Rabbit anti-Vimentin (1: 1000)	Cell Signaling Technology	5741T
Rabbit anti-Zo-1 (1: 1000)	Cell Signaling Technology	8193
Rabbit anti-N-cadherin (1: 1000)	Cell Signaling Technology	13116
Rabbit anti-Slug (1: 1000)	Cell Signaling Technology	9585
Rabbit anti-IL-6 (1: 1000)	Cell Signaling Technology	12153
Rabbit anti-Ki-67 (IHC)	Ventana	790-4286
Mouse anti-E-cadherin (IHC)	Ventana	790-4497
Mouse anti-Vimentin (IHC)	Ventana	790-2917
Rabbit anti-IL-6 (IHC)	Thermo Fisher Scientific	21865-1-AP
Alexa Fluor 647 Goat anti-mouse IgG (1:200)	Invitrogen	A21235
HRP-conjugated Goat anti-Mouse IgG (1:2000)	EMD Millipore	AP124P
HRP-conjugated Goat anti-Rabbit IgG (1:2000)	EMD Millipore	AP132P
**Chemicals, neutralizing antibodies and recombinant proteins**		
Cell lysis buffer	Cell Signaling Technology	9803
Protease inhibitor cocktail	Roche	11 836 153 001
Lipofectamine 3000	Invitrogen	L3000-008
Puromycin	Gibco	A11138-03
Polybrene	Santa Cruz Biotechnology	sc-134220
Neutralizing antibody anti-human IL-6	R&D systems	AB-206-NA
Normal Goat IgG control	R&D systems	AB-108-C
Recombinant human IL-6	R&D systems	206-IL
Recombinant human insulin	Gibco	12585-014
Recombinant human basic FGF	Gibco	13256029
Recombinant human EGF	Sigma	E5036
Matrigel matrix	Corning	354234
MethoCult™ H4100	Stem Cell Technologies	04100
Collagen type I rat tail	BD Biosciences	354236
D-Luciferin potassium salt	PerkinElmer	122799
ProLong™ Diamond Antifade Mountant with DAPI	Life Technologies	P36962
Tri reagent	Molecular Research Center	TR118
RevertAid First Strand cDNA Synthesis Kit	Thermo Fisher Scientific	K1622
SYBR™ Select Master Mix	Thermo Fisher Scientific	4472908
**Commercial assays**		
Human cytokine array C1000	RayBiotech	AAH-CYT-1000

Human IL-6 ELISA	R&D systems	QK206
Cell Counting Kit-8 (CCK-8)	Dojindo Laboratories	CK04
ultraView universal DAB detection kit (IHC)	Ventana	760-500
**Plasmids**		
MSI2 CRISPR-Cas9	GenScript	SC1805
MSI2_OHu09006D_pcDNA3.1	GenScript	SC1200
pLentiCRISPR v2	GenScript	SC1818
pCMV-VSV-G	Addgene	8454
pCMV-dR8.2 dvpr	Addgene	8455
UBC-RFP-T2A-Luciferase	SBI System Biosciences	BLIV200PA-1
**Others**		
Transwell 8.0 µm pore size; polycarbonate membrane	Corning	3422
IVIS Lumina II in Vivo Imaging system	PerkinElmer	N/A
Inverted fluorescence/phase-contrast microscope; BZ-X700/BZ-X710	Keyence	N/A
ELx800 absorbance microplate reader	BioTek	N/A
Amersham™ Imager 680	GE Healthcare Bio-sciences	N/A
NanoDrop 2000 spectrophotometer	Thermo Fisher Scientific	N/A
CFX384 Touch™ Real-Time PCR Detection System	Bio-Rad	N/A

Table S2. Databases used for bioinformatic analyses.

GEO accession	Analysis	Platform		Probe	Source
GSE19188	GEO2R	GPL570	Affymetrix Human Genome U133 Plus 2.0 Array	*MSI2*: 1552364_s_at	[1]
GSE31552	GEO2R	GPL6244	Affymetrix Human Gene 1.0 ST Array	*MSI2*: 8008682	[2]
GSE31210	GEO2R	GPL570	Affymetrix Human Genome U133 Plus 2.0 Array	*MSI2*: 1552364_s_at	[3, 4]
GSE118370	GEO2R	GPL570	Affymetrix Human Genome U133 Plus 2.0 Array	*MSI2*: 1552364_s_at	[5]
GSE115002	GEO2R	GPL13497	Agilent-026652 Whole Human Genome Microarray 4x44K v2	*MSI2*: A_23_P369479	[6]
GSE30219	GEO2R	GPL570	Affymetrix Human Genome U133 Plus 2.0 Array	*MSI2*: 1552364_s_at	[7]
GSE32537	GEO2R	GPL6244	Affymetrix Human Gene 1.0 ST Array	*MSI2*: 8008682; *ACTA2*: 7934906	[8]

GSE10667	GEO2R	GPL4133	Agilent-014850 Whole Human Genome Microarray 4x44K G4112F	*MSI2*: 30788; *ACTA2*: 10395	[9–12]
GSE22874	GEO2R	GPL5175	Affymetrix Human Exon 1.0 ST Array	*MSI2*: 3728147	[13]

Table S3. Primer sequences for quantitative real-time PCR (RT-qPCR).

Gene	Forward primer (5'–3')	Reverse primer (5'–3')
MSI2 [14]	GATCCCACTACGAAACGCTCCA	GTCTGCGAACGTGACGAAACC
IL6	CCACCGGGAACGAAAGAGAA	TCACCAGGCAAGTCTCCTCA
JAK2	GTCGCCCGATCTGTGTAGC	TTTCAGAACATTTGCCGTCGC
STAT3	CTGCCCCATACCTGAAGACC	AGGTGAGGGACTCAAACTGC
NFKB1	GCAGATGGCCCATACCTTCA	GCATTGGGGGCTTTACTGTC
NFKB2	CTTTAGCGGACAGCGCCT	TAGGGGCCATCAGCTGTTTCT
P65	GGCGAATGGCTCGTCTGTAG	CCAGGTTCTGGAAACTGTGGA
REL	ACCCAATTTATGACAACCGTGC	CCAGGTCTTGGTCTCTCAGGA
GAPDH	AGCCACATCGCTCAGACAC	GCCCAATACGACCAAATCC

Additional References

1. Hou J, Aerts J, den Hamer B, van Ijcken W, den Bakker M, Riegman P, et al. Gene expression-based classification of non-small cell lung carcinomas and survival prediction. PLoS One. 2010;5(4):e10312.
2. Lin J, Marquardt G, Mullapudi N, Wang T, Han W, Shi M, et al. Lung cancer transcriptomes refined with laser capture microdissection. Am J Pathol. 2014;184(11):2868–84.
3. Okayama H, Kohno T, Ishii Y, Shimada Y, Shiraishi K, Iwakawa R, et al. Identification of genes upregulated in ALK-positive and EGFR/KRAS/ALK-negative lung adenocarcinomas. Cancer Res. 2012;72(1):100–11.
4. Yamauchi M, Yamaguchi R, Nakata A, Kohno T, Nagasaki M, Shimamura T, et al. Epidermal growth factor receptor tyrosine kinase defines critical prognostic genes of stage I lung adenocarcinoma. PLoS One. 2012;7(9):e43923.
5. Xu L, Lu C, Huang Y, Zhou J, Wang X, Liu C, et al. SPINK1 promotes cell growth and metastasis of lung adenocarcinoma and acts as a novel prognostic biomarker. BMB Rep. 2018;51(12):648–53.
6. Cui Y, Fang W, Li C, Tang K, Zhang J, Lei Y, et al. Development and validation of a novel signature to predict overall survival in "Driver Gene-negative" lung adenocarcinoma (LUAD): results of a multicenter study. Clin Cancer Res. 2019;25(5):1546–56.
7. Rousseaux S, Debernardi A, Jacquiau B, Vitte AL, Vesin A, Nagy-Mignotte H, et al. Ectopic activation of germline and placental genes identifies aggressive metastasis-prone lung cancers. Sci Transl Med. 2013;5(186):186ra66.

8. Yang IV, Coldren CD, Leach SM, Seibold MA, Murphy E, Lin J, et al. Expression of cilium-associated genes defines novel molecular subtypes of idiopathic pulmonary fibrosis. Thorax. 2013;68(12):1114–21.

9. Konishi K, Gibson KF, Lindell KO, Richards TJ, Zhang Y, Dhir R, et al. Gene expression profiles of acute exacerbations of idiopathic pulmonary fibrosis. Am J Respir Crit Care Med. 2009;180(2):167–75.

10. Rosas IO, Richards TJ, Konishi K, Zhang Y, Gibson K, Lokshin AE, et al. MMP1 and MMP7 as potential peripheral blood biomarkers in idiopathic pulmonary fibrosis. PLoS Med. 2008;5(4):e93.

11. Vuga LJ, Milosevic J, Pandit K, Ben-Yehudah A, Chu Y, Richards T, et al. Cartilage oligomeric matrix protein in idiopathic pulmonary fibrosis. PLoS One. 2013;8(12):e83120.

12. Yamashita CM, Dolgonos L, Zemans RL, Young SK, Robertson J, Briones N, et al. Matrix metalloproteinase 3 is a mediator of pulmonary fibrosis. Am J Pathol. 2011;179(4):1733–45.

13. Navab R, Strumpf D, Bandarchi B, Zhu CQ, Pintilie M, Ramnarine VR, et al. Prognostic gene-expression signature of carcinoma-associated fibroblasts in non-small cell lung cancer. Proc Natl Acad Sci U S A. 2011;108(17):7160–5.

14. Zhang X, Su K, Liu Y, Zhu D, Pan Y, Ke X et al. Small molecule palmatine targeting Musashi-2 in colorectal cancer. Front Pharmacol. 2022;12.
