# Supplementary material for: Musashi-2 in cancer-associated fibroblasts promotes non-small cell lung cancer metastasis through paracrine IL-6-driven epithelial-mesenchymal transition
Source: Cell Biosci. 2023 Nov 8;13:205. doi: 10.1186/s13578-023-01158-5 (PMC10631049; doi:10.1186/s13578-023-01158-5)
Supplement: Supplementary file 2 — Additional file 2: Fig. S1. MSI2 is highly expressed in the CAFs derived from patients with advanced NSCLC. Fig. S2. CAFs exhibited higher α-SMA and MSI2 than NFs. Fig. S3. MSI2 deficiency in CAFs has no effect on NSCLC cell proliferation and clonogenic growth. Fig. S4. MSI2 deficiency in CAFs inhibits NSCLC cell migration. Fig. S5. Treatment of NSCLC cells with CM from Ctrl and gMSI2 CAFs has minimal effect on cell proliferation. Fig. S6 MSI2 in CAFs modulates NSCLC cell motility. Fig. S7. MSI2 in CAFs modulates its cell motility. Fig. S8. MSI2 in CAFs promotes NSCLC metastasis in nude mice. Fig. S9. Cytokine secretion from Ctrl and gMSI2 CAFs. Fig. S10. IHC analysis of IL-6 in primary tumors obtained from mice bearing NCIH292 cells with Ctrl or gMSI2 CAFs. Fig. S11. Downregulation of multitude of genes involved in JAK2/STAT3 and NF- κB signaling pathways upon MSI2 depletion in CAFs. Fig. S12. IL-6 restores the inhibitory effect of MSI2-deficient CAFs on NSCLC cell migration and invasion. Fig. S13. Ctrl and gMSI2 CAF-CM with control IgG Ab, neutralizing anti-IL-6 Ab, or recombinant IL-6 have no appreciable effect on NSCLC cell proliferation. Fig. S14. MSI2 in CAFs mediates EMT activation in NSCLC via paracrine IL-6. Fig. S15. IL-6 activates EMT in NSCLC cells. Fig. S16. Expression level of MSI2 is positively correlated with ACTA2. [file 13578_2023_1158_MOESM2_ESM.pdf]

Additional file 2: Additional Figures

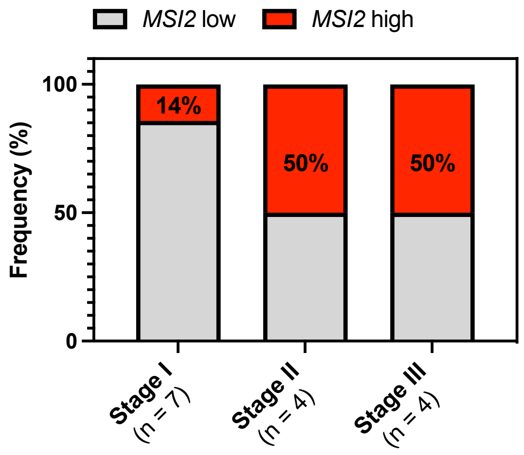

Additional Fig. S1 *MSI2* is highly expressed in the CAFs derived from patients with advanced NSCLC. *MSI2* expression and clinical characteristics of patients were obtained from Gene Expression Omnibus (accession number GSE22874). Determination of CAFs with *MSI2* high and *MSI2* low was based on the comparative analysis between *MSI2* expression in CAFs and its pair-matched NFs from the same patient. CAFs exhibiting higher *MSI2* than those of NFs are classified as CAFs with *MSI2* high, and vice versa. The frequency of CAFs with *MSI2* high and *MSI2* low was plotted. CAF, cancer-associated fibroblast; NSCLC, non-small cell lung cancer; NF, normal fibroblast.

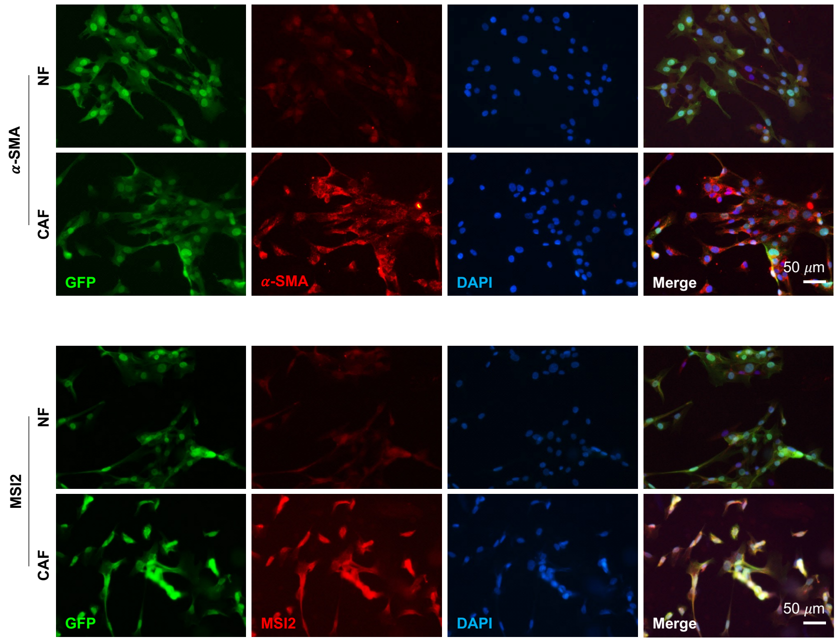

Additional Fig. S2 CAFs exhibited higher α-SMA and MSI2 than NFs. CAFs and NFs were genetically labeled with GFP (green), immunostained for α-SMA (upper, red) or MSI2 (lower, red), and examined under fluorescence microscope. Nuclei were counterstained with DAPI (blue). Images were taken pairwise with the same instrumental setting. Scale bar = 50 μm.

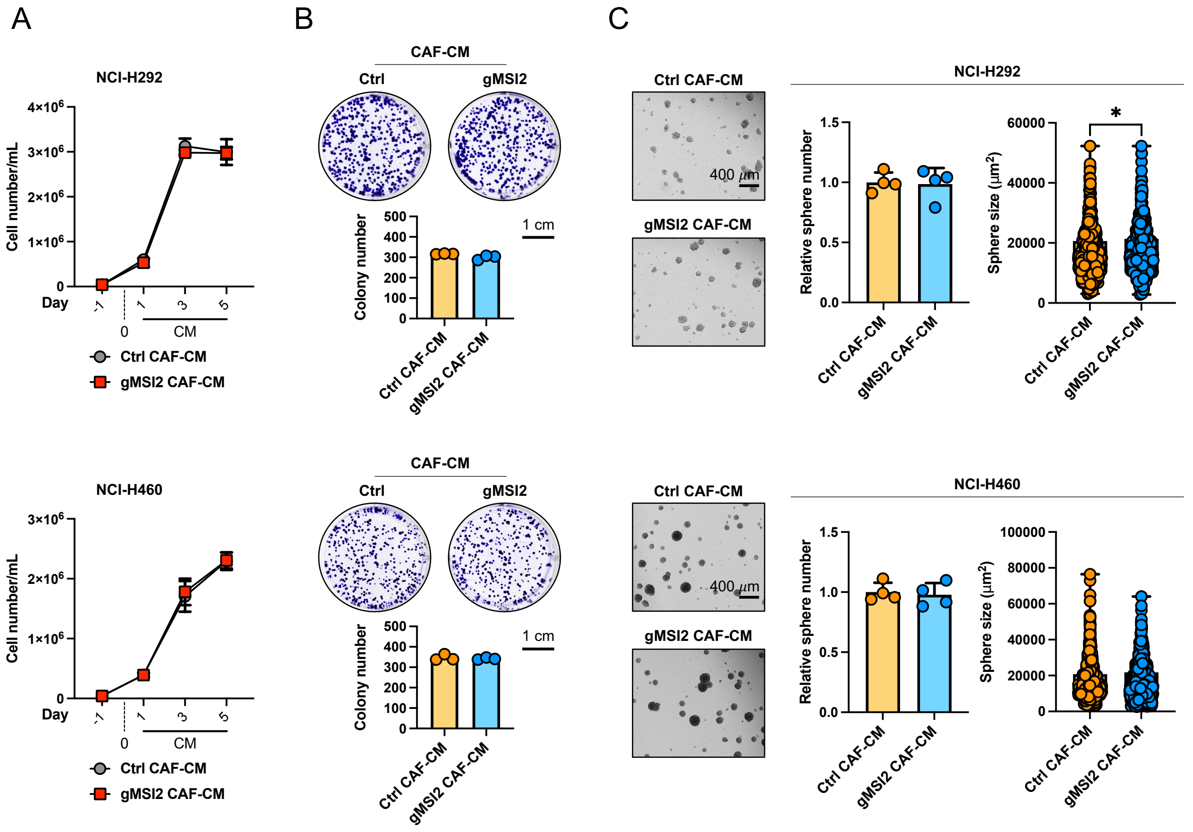

Additional Fig. S3 MSI2 deficiency in CAFs has no effect on NSCLC cell proliferation and clonogenic growth. **A** Proliferation assay of NCI-H292 (upper) and NCI-H460 (lower) NSCLC cells. Cells were seeded at a density of 4×10^4 (Day -1) and cultured in CM from Ctrl or gMSI2 CAFs the following day (Day 0). CM, conditioned media. Cells were counted by trypan blue exclusion at day 1, 3, and 5. Data are presented as mean ± SD (n = 4); two-tailed Student's *t*-test. **B** Colony formation assay of NCI-H292 (upper) and NCI-H460 (lower) NSCLC cells. One week after culturing with Ctrl CAF-CM or gMSI2 CAF-CM, colonies were stained with crystal violet to visualize and number. Colonies were quantified below using ImageJ. Representative whole wells are shown. Scale bar = 1 cm. Data are presented as mean ± SD (n = 3); two-tailed Student's *t*-test. **C** Sphere-forming assay of NCI-H292 (upper) and NCI-H460 (lower) NSCLC cells was performed after one week of culturing with Ctrl CAF-CM or gMSI2 CAF-CM in MethoCult^{TM}. Representative bright field micrographs are shown (left). Scale bar = 400 μm. Relative number of spheres to Ctrl CAF-CM was quantified. Data are presented as

mean ± SD (n = 4); two-tailed Student's *t*-test. Size of spheres was measured. Data are presented as mean ± SD (n > 600 spheres per group; r = 4); $^{*}P < 0.05$ versus Ctrl CAF-CM; two-tailed Student's *t*-test. r = biological replicates.

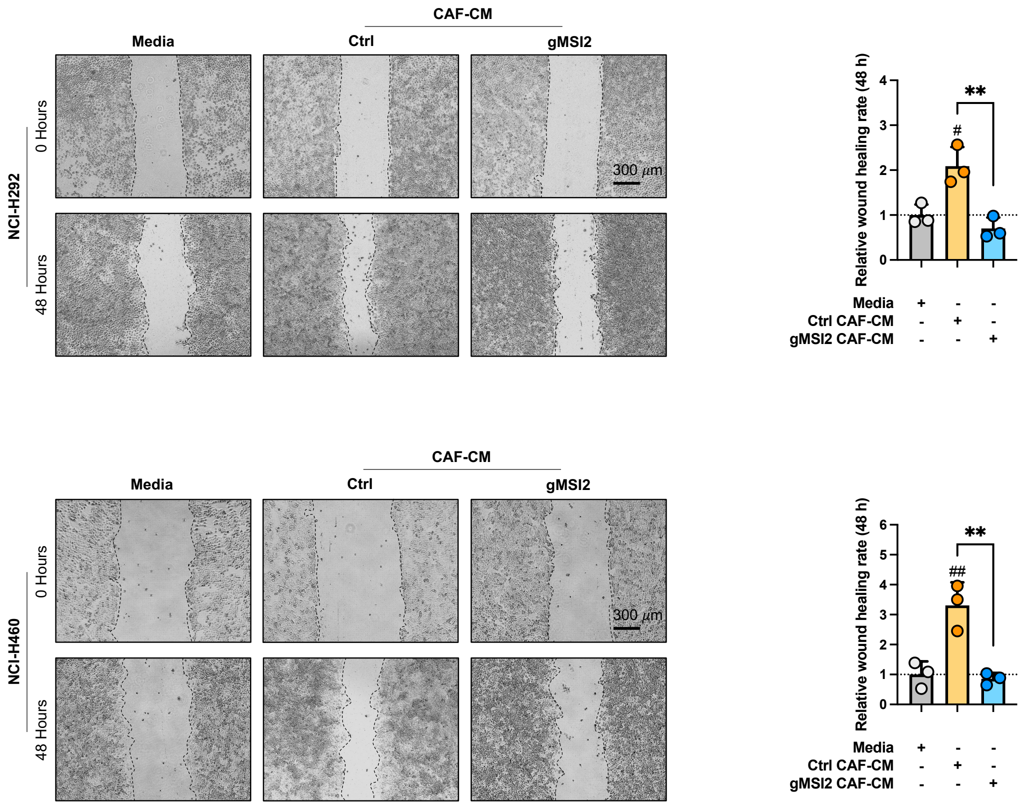

Additional Fig. S4 MSI2 deficiency in CAFs inhibits NSCLC cell migration. Wound-healing assays were performed in NCI-H292 (upper) and NCI-H460 (lower) NSCLC cells with or without CM from Ctrl or gMSI2 CAFs. Representative micrographs of scratch gap are shown and analyzed (left) at 0- or 48-h time points. Scale bar = 300 μm. Bar graph depicts relative wound healing rate (right). Data are presented as mean ± SD (n = 3); $^{\#}P < 0.05$, $^{\#\#}P < 0.01$ versus media; $^{**}P < 0.01$, versus Ctrl CAF-CM; one-way ANOVA and Tukey's multiple comparison test.

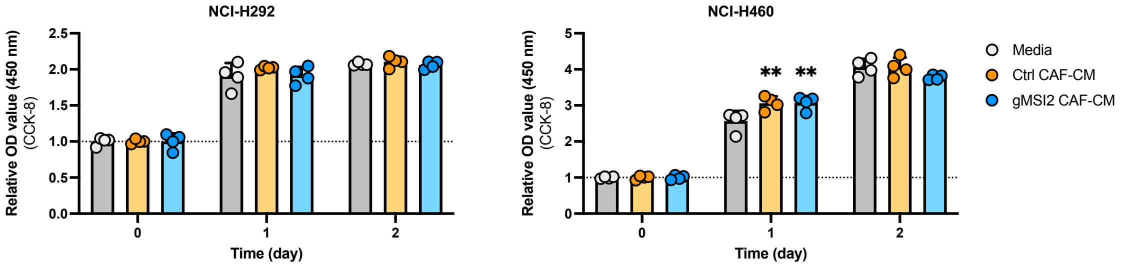

Additional Fig. S5 Treatment of NSCLC cells with CM from Ctrl and gMSI2 CAFs has minimal effect on cell proliferation. Experimental design is depicted in Fig. 2C. NCI-H292 (left) and NCI-H460 (right) NSCLC cells were seeded into 96-well plates, and cell proliferation was measured at day 0, 1, 2, and expressed relative to day 0 using CCK-8 assay. Data are presented as mean ± SD (n = 4); $^{**}P < 0.01$ versus media; two-way ANOVA and Tukey's multiple comparison test.

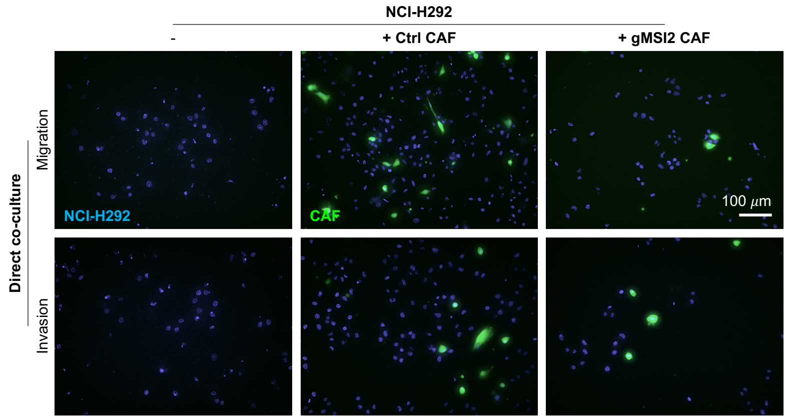

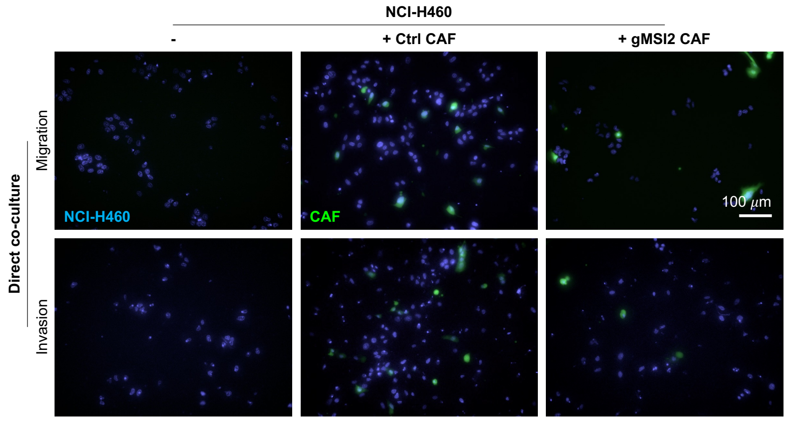

Additional Fig. S6 MSI2 in CAFs modulates NSCLC cell motility. Transwell assay showing that NCI-H292 (upper) and NCI-H460 (lower) NSCLC cells in combination with GFP-labeled gMSI2 CAFs (ratio 1:1) exhibited decreased cell migration and invasion activities. Representative merged micrographs of migrating/invading cells stained with Hoechst 33342 are shown, related to Fig. 2E. Scale bar = 100 μm. Green fluorescence, CAFs.

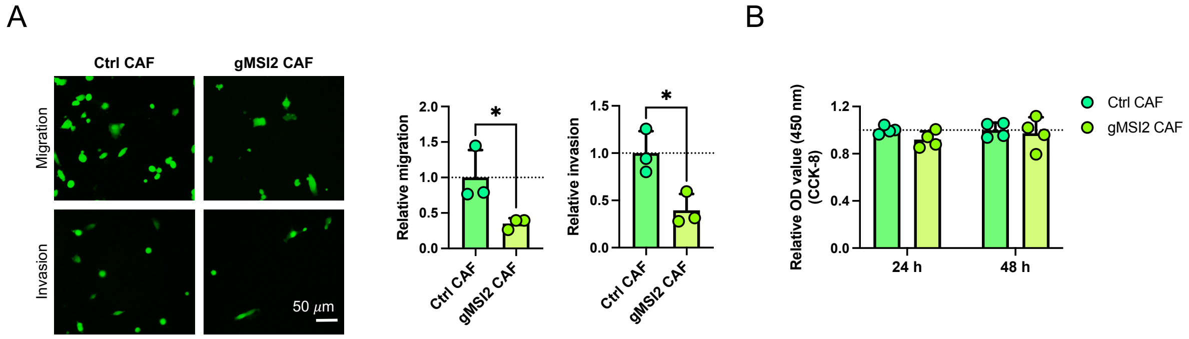

Additional Fig. S7 MSI2 in CAFs modulates its cell motility. Transwell migration and invasion assays of GFP-labeled gMSI2 CAFs and Ctrl CAFs were performed without tumor co-culture. **A** (left) Representative micrographs of migrating/invading cells are shown. Scale bar = 50 μm. (right) Bar graph depicts relative cell migration and invasion. Data are presented as mean ± SD (n = 4); $^{*}P < 0.05$ versus Ctrl CAFs; two-tailed Student's *t*-test. **B** Depletion of MSI2 in CAFs did not affect cell viability as evaluated by CCK-8 assay. Data are presented as mean ± SD (n = 4); two-tailed Student's *t*-test.

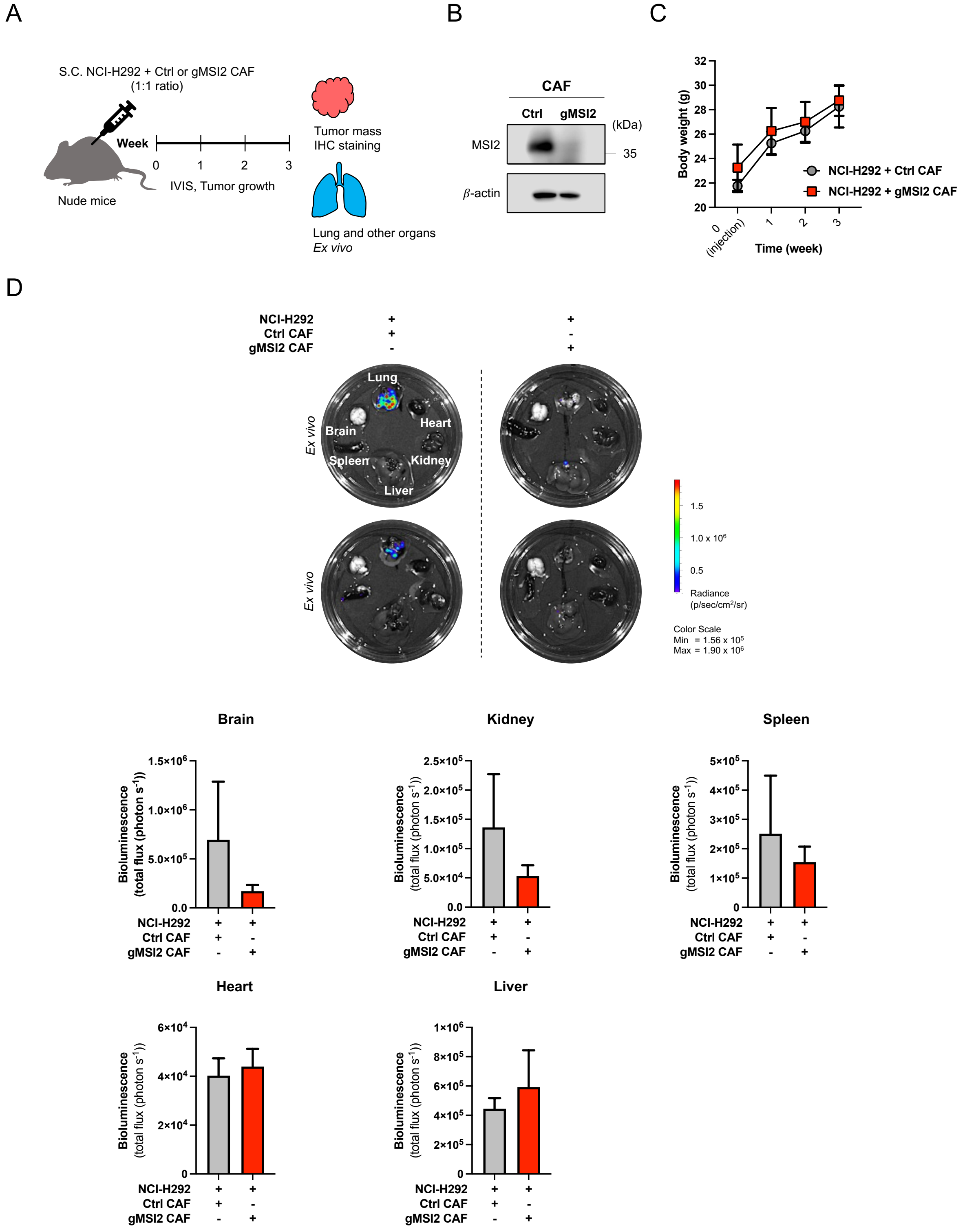

Additional Fig. S8 MSI2 in CAFs promotes NSCLC metastasis in nude mice. **A** Experimental design, related to Fig. 3. NCI-H292 NSCLC cells labeled with luciferase were combined with Ctrl CAFs or gMSI2 CAFs and injected subcutaneously into male NU/J mice. IVIS was utilized

to monitor tumor progression, and its size was recorded weekly. On week 3, all mice were euthanized, and primary tumors and indicated organs were collected and analyzed. **B** Western blotting confirming that gMSI2 CAFs was deficient in MSI2 protein expression prior to in vivo mouse studies. β-actin was used as a loading control. **C** Body weight of mice over three weeks. Data are presented as mean ± SD (n = 4); two-tailed Student's *t*-test. **D** To assess metastatic burden, organs were collected for ex vivo studies according to the PerkinElmer protocol. (upper) Representative ex vivo images of isolated internal organs, including lung, brain, kidney, spleen, heart, and liver, are shown. (lower) Plots showing quantified bioluminescence signals obtained from organs of mice. Data are presented as mean ± S.E.M. (n = 3 or 4); Mann-Whitney test, two-sided.

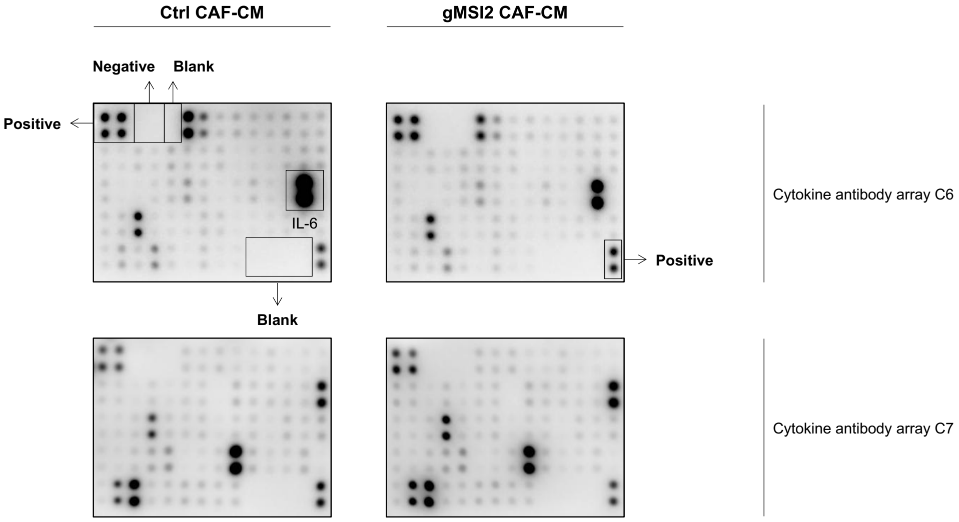

Additional Fig. S9 Cytokine secretion from Ctrl and gMSI2 CAFs. Representative images of broad human cytokine array analysis in CM of Ctrl and gMSI2 CAFs are shown, related to Fig. 4A and B. The IL-6 location is highlighted.

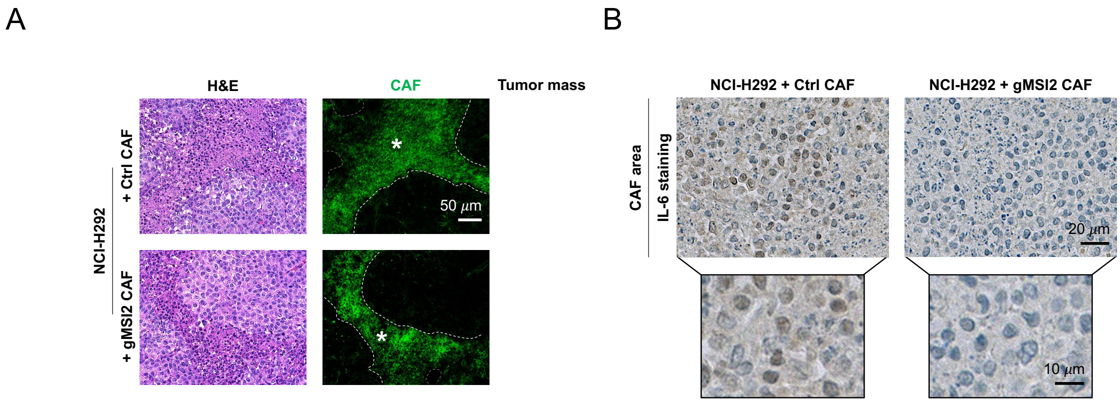

Additional Fig. S10 IHC analysis of IL-6 in primary tumors obtained from mice bearing NCI-H292 cells with Ctrl or gMSI2 CAFs. **A** H&E micrographs of primary tumors confirmed the presence Ctrl or gMSI2 CAFs. GFP fluorescence was used to mark the CAF area (green, asterisk). Scale bar = 50 µm. **B** Representative IHC staining of IL-6 in CAF area. Scale bars are as indicated.

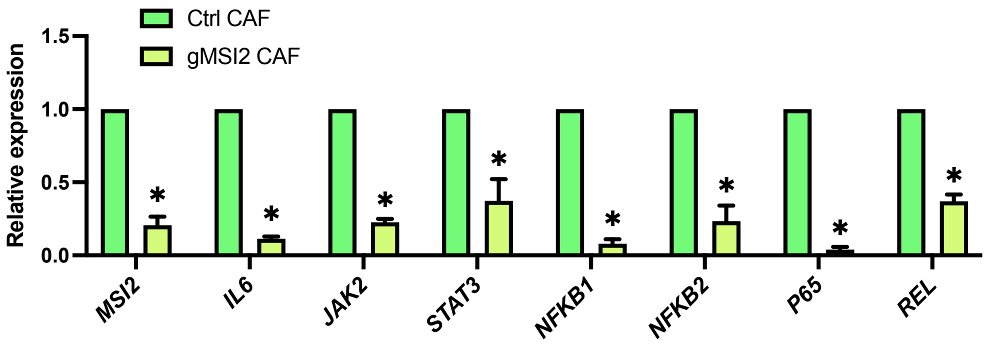

Additional Fig. S11 Downregulation of multitude of genes involved in JAK2/STAT3 and NF-κB signaling pathways upon MSI2 depletion in CAFs. RT-qPCR analysis of *MSI2*, *IL-6*, *JAK2*, *STAT3*, *NFKB1*, *NFKB2*, *P65*, and *REL*. *GAPDH* served as the internal control. Data are presented as mean ± SD (n = 3); $^{*}P < 0.05$ versus Ctrl CAFs; two-tailed Student's *t*-test.

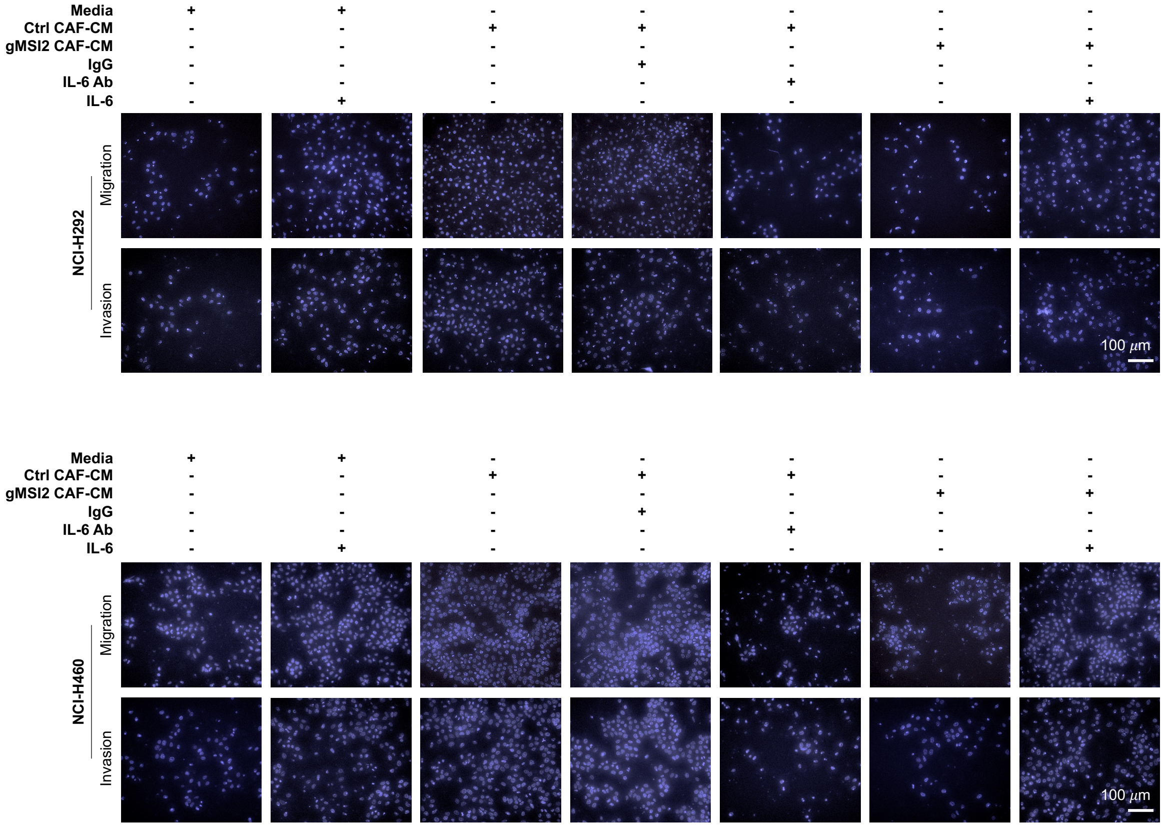

Additional Fig. S12 IL-6 restores the inhibitory effect of MSI2-deficient CAFs on NSCLC cell migration and invasion. Transwell assay was performed to evaluate cell migration and invasion. From left to right, NCI-H292 (upper) and NCI-H460 (lower) NSCLC cells were cultured in either Ctrl CAF-CM or gMSI2 CAF-CM supplemented with control IgG Ab, neutralizing anti-IL-6 Ab, or recombinant IL-6, as indicated. Representative micrographs of migrating/invading cells stained with Hoechst 33342 are shown, related to Fig. 5A. Scale bar = 100 μm.

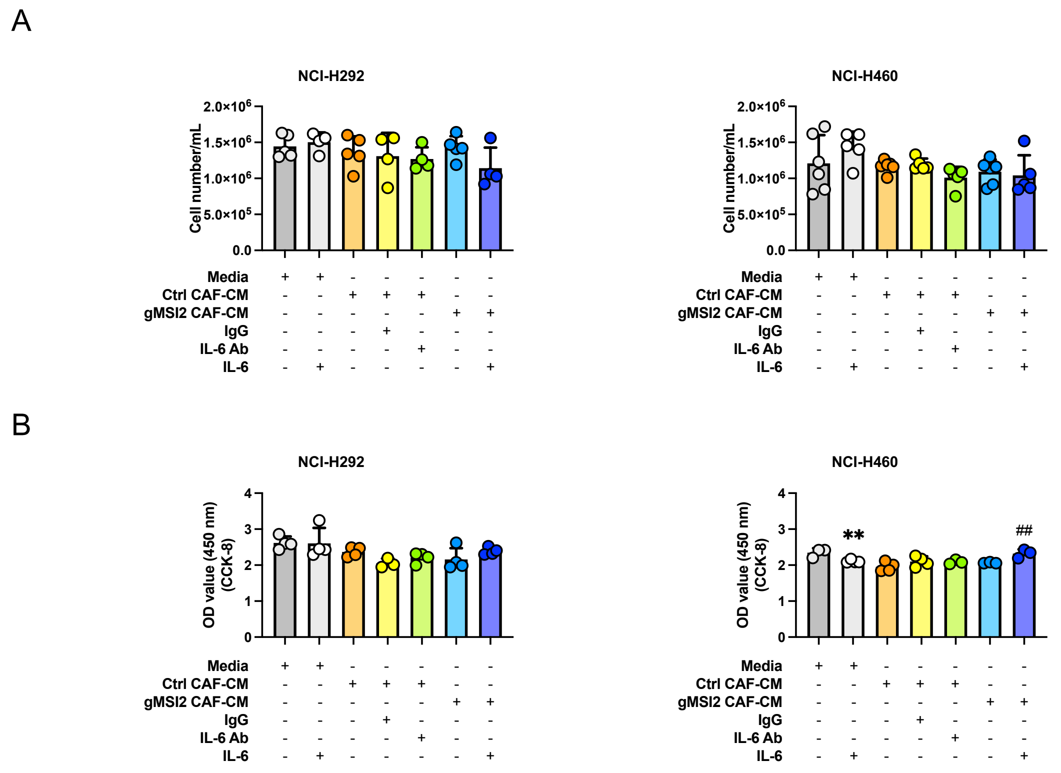

Additional Fig. S13 Ctrl and gMSI2 CAF-CM with control IgG Ab, neutralizing anti-IL-6 Ab, or recombinant IL-6 have no appreciable effect on NSCLC cell proliferation. **A** NCI-H292 (left) and NCI-H460 (right) NSCLC cells were incubated with Ctrl CAF-CM or gMSI2 CAF-CM containing the indicated cytokine or antibody for up to 48 h. Cells were counted by trypan blue exclusion. Data are presented as mean ± SD (n = 4–6); one-way ANOVA and Tukey's multiple comparison test. Ab, antibody. **B** Experimental design is shown in Fig. 2C. NCI-H292 (left) and NCI-H460 (right) NSCLC cells were seeded into 96-well plates, and cell proliferation was measured at day 0, 1, 2, and expressed relative to day 0 using CCK-8 assay. Data are presented as mean ± SD (n = 3 or 4); $^{**}P < 0.01$ versus media; $^{\#\#}P < 0.01$ versus Ctrl CAF-CM; one-way ANOVA and Tukey's multiple comparison test.

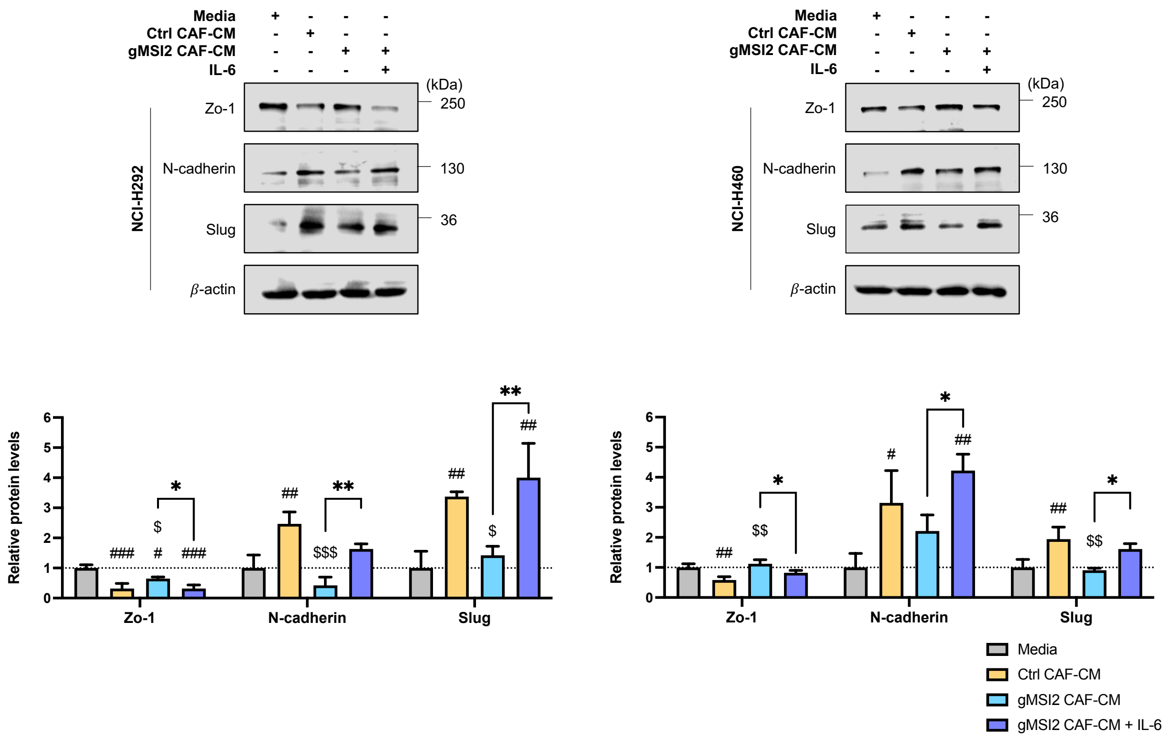

Additional Fig. S14 MSI2 in CAFs mediates EMT activation in NSCLC via paracrine IL-6. EMT markers were analyzed by Western blotting in NSCLC NCI-H292 (left) and NCI-H460 (right) cells following Ctrl CAF-CM or gMSI2 CAF-CM exposure in the presence or absence of IL-6 for 48 h. Additionally, a rescue experiment was conducted by supplementing gMSI2 CAF-CM with IL-6 for 48 h. β-actin was used as a loading control. Data are mean ± SD (n = 3); $^{\#}P < 0.05$, $^{\#\#}P < 0.01$, $^{\#\#\#}P < 0.001$ versus media; $^{\$}P < 0.05$, $^{\$\$}P < 0.01$, $^{\$\$\$}P < 0.001$ versus Ctrl CAF-CM; $^{*}P < 0.05$, $^{**}P < 0.01$ versus gMSI2 CAF-CM; one-way ANOVA with Tukey's multiple comparison test.

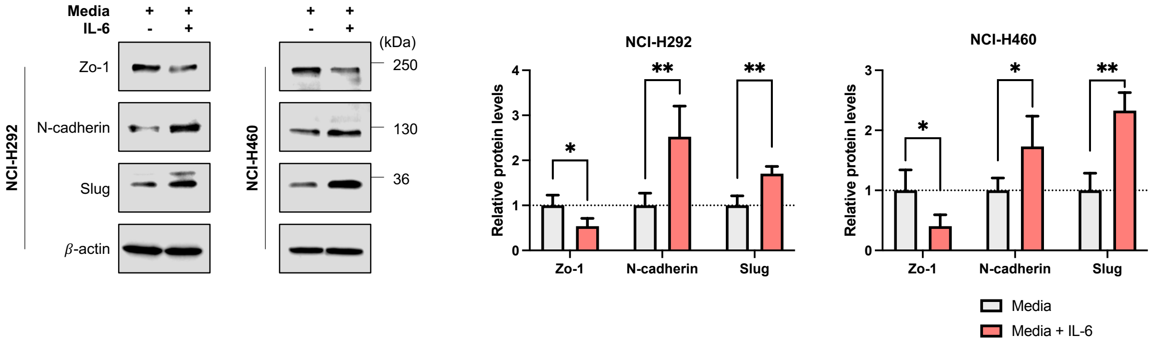

Additional Fig. S15 IL-6 activates EMT in NSCLC cells. NSCLC NCI-H292 (left) and NCI-H460 (right) cells were treated with human recombinant IL-6 for 48 h, and EMT markers were evaluated by Western blotting. β-actin was used as a loading control. Data are presented as mean ± SD (n = 3 or 4); $^{*}P < 0.05$, $^{**}P < 0.01$ versus media; two-tailed Student's *t*-test.

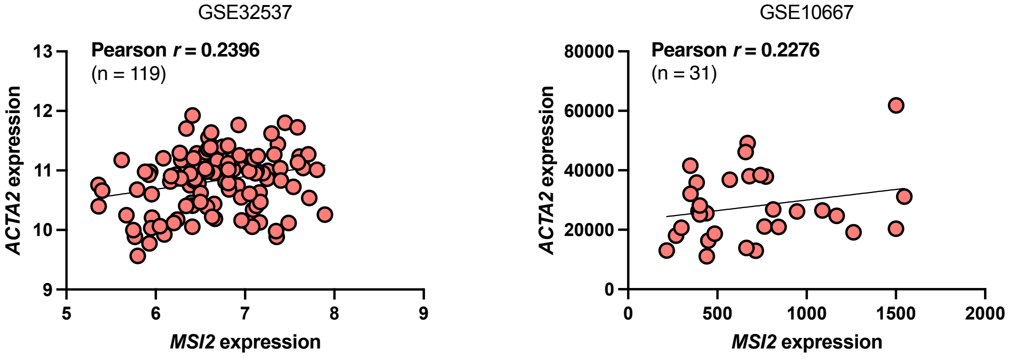

Additional Fig. S16 Expression level of *MSI2* is positively correlated with *ACTA2*. Correlation analysis between *MSI2* expression and *ACTA2* expression (encoding α-SMA) from two independent human IPF/UIP datasets from GSE32537 and GSE10667. Correlation coefficient (*r*) is indicated on the plot.
